# Supplementary material for: Adolescent’s descriptions of fatigue, fluctuation and payback in chronic fatigue syndrome/myalgic encephalopathy (CFS/ME): interviews with adolescents and parents
Source: BMJ Paediatr Open. 2018 Dec 4;2(1):e000281. doi: 10.1136/bmjpo-2018-000281 (PMC6307594; doi:10.1136/bmjpo-2018-000281)
Supplement: Supplementary file 1 [file bmjpo-2018-000281supp001.docx]

**APPENDIX A: Outcomes in Paediatric CFS/ME**

**Extract from Young Person Topic Guide**

**Introduction**

1. **How does having CFS/ME affect your life?**

Prompts: Can you tell me more about X, what is it about X, can you describe that…

**Recovery**

1. **How do you know when you are feeling worse?**
2. **How do you know when you are feeling better?**
3. **What things if any stay the same whether you are better or worse?**
4. **How would you know if you were completely better again?**

Prompts: What would need to improve for you to say that you are better, by how much?

What would you like to do that you are unable to now?

What things can you cope with if they don’t improve/ return to normal?

**Outcome Domains**

*Activity: Present YP with outcome domain cards. Add any domains to the blank cards the YP may have spontaneously brought up. Get YP to select the areas most affected by CFS/ME and rank them in order of importance as to what is most important to improve.*

*Top 5 domains- explore dimensions of domains (frequency/ severity/ duration/ satisfaction)*

*Prompts e.g. what symptoms bother you most, what is important about school.*

*[Take a photo of order].*

1. **Pick out the top issues/areas of your life most affected by CFS/ME (upto 10).**
2. **Imagine you were able to improve these areas, rank these in order of what you feel is most important to improve, put the areas at the top you would most like to improve.**

Tiredness

Symptoms (pain, headaches, feeling sick, brain fog)

Sleep problems

Daily activities (getting up, getting dressed, going out)

Payback & crashing (tired after activity)

Fluctuation (changing symptoms- good day vs. bad day)

School (attendance, concentrating, keeping up with work)

Activities & hobbies (sports, clubs)

Spending time with friends

Family activities

Mood (feeling down, worrying)

How you feel about yourself (confidence, personality)

Your future (GCSEs, college, jobs)

Independence (doing things without your parents)

Seeing your boyfriend/girlfriend

1. **Why have you ranked them in that order?**

**Top 5 domains**- explore dimensions of domains (frequency/ severity/ duration/ satisfaction)

- Prompts e.g. what symptoms bother you most, what is important about school.

1. **How might your answer have been different a year ago? [Pick the cards].**

- Why were they important?
- In what ways has it changed/ improved?

**Ending question**

1. **Is there anything else about having CFS/ME that you feel is important to you?**
