# Supplementary material for: Adolescent’s descriptions of fatigue, fluctuation and payback in chronic fatigue syndrome/myalgic encephalopathy (CFS/ME): interviews with adolescents and parents
Source: BMJ Paediatr Open. 2018 Dec 4;2(1):e000281. doi: 10.1136/bmjpo-2018-000281 (PMC6307594; doi:10.1136/bmjpo-2018-000281)
Supplement: Supplementary file 2 [file bmjpo-2018-000281supp002.docx]

**APPENDIX B: Thematic Framework**

| **Overarching themes (deductive)**  *From card ranking exercise* | **Subthemes (inductive)** |
| --- | --- |
| **FATIGUE AND SYMPTOMS** | **Sleep**  Problems at night  Sleeping during the day (moderate)  **Tiredness and lack of energy**  Tired all the time (duration)  Feeling quite worn out (severity)  **Memory and concentrations**  **Pain**  Muscle pain and aches  Headaches  Sore throat  Stomach ache  Pain quite bad (severity of pain)  Sick-nausea-no appetite  Dizziness  Sensitivity to light  Feeling cold  Feel horrible, ill  Feeling weak  **Dimensions of Symptoms**  **Only one symptom vs. collection of symptoms**  **Frequency of symptoms (all the time, changes)**  **Severity of symptoms (its really bad)** |
| **FLUCTUATION** | **Bad day**  Symptoms get worse on bad days  Cant do anything  More tired  Other people notice physical signs-pale  Get more payback if do things on a bad day  **Good day**  Feel more energetic on better days  Can do more  Feel happier  More talkative, sociable  No-less symptoms  Feel better in the morning-had more sleep  Go to school  Less tired |
| **PAYBACK** | Trying to do more tires me out-be completely wiped-cant do anything the next day  Have to plan activities  Can do things e.g. go into town but get payback  Symptoms get worse |
| **IMPACT ON PHYSICAL FUNCTIONING** | **Limiting leisure activities**  Cant go out for too long  Cant do what you want to  **Problems with daily activities-getting dressed, bathing**  Problems walking  Problems climbing stairs  Need to use wheelchair  **Tiredness-cant wake up-get going (sedentary)**  Slumped on the couch  Cant really do anything (sit in bed or on the sofa) |
