# Supplementary material for: Adolescent’s descriptions of fatigue, fluctuation and payback in chronic fatigue syndrome/myalgic encephalopathy (CFS/ME): interviews with adolescents and parents
Source: BMJ Paediatr Open. 2018 Dec 4;2(1):e000281. doi: 10.1136/bmjpo-2018-000281 (PMC6307594; doi:10.1136/bmjpo-2018-000281)
Supplement: Author's manuscript [file bmjpo-2018-000281.draft_revisions.pdf]

BMJ Paediatrics Open

BMJ Paediatrics Open is committed to open peer review. As part of this commitment we make the peer review history of every article we publish publicly available.

When an article is published we post the peer reviewers' comments and the authors' responses online. We also post the versions of the paper that were used during peer review. These are the versions that the peer review comments apply to.

The versions of the paper that follow are the versions that were submitted during the peer review process. They are not the versions of record or the final published versions. They should not be cited or distributed as the published version of this manuscript.

BMJ Paediatrics Open is an open access journal and the full, final, typeset and author-corrected version of record of the manuscript is available on our site with no access controls, subscription charges or pay-per-view fees (http://bmjpaedsopen.bmj.com).

If you have any questions on BMJ Paediatrics Open's open peer review process please email info.bmjpo@bmj.com

BMJ Paediatrics Open

Confidential: For Review Only

Adolescent's descriptions of fatigue, fluctuation and payback in Chronic Fatigue Syndrome/Myalgic Encephalopathy (CFS/ME): A qualitative study

Journal:	*BMJ Paediatrics Open*
Manuscript ID	bmjpo-2018-000281
Article Type:	Original article
Date Submitted by the Author:	01-Mar-2018
Complete List of Authors:	Parslow, Roxanne; University of Bristol, Centre for Child and Adolescent Health<br>Anderson, Nina; University of Bristol, Centre for Child and Adolescent Health<br>Byrne, Danielle; University of Bristol, Centre for Child and Adolescent Health<br>Shaw, Alison; University of Bristol, 2. Centre for Primary Care Research<br>Haywood, Kirstie; University of Warwick, Warwick Research in Nursing<br>Crawley, Esther; University of Bristol School of Social and Community Medicine, Centre for Child and Adolescent Health
Keywords:	Chronic Fatigue Syndrome, Adolescent Health, Qualitative research, Patient perspective

SCHOLARONE™
Manuscripts

BMJ Paediatrics Open

Adolescent's descriptions of fatigue, fluctuation and payback in Chronic Fatigue Syndrome/Myalgic Encephalopathy (CFS/ME): A qualitative study

Roxanne, M. Parslow[1§], Nina Anderson[1], Danielle Byrne[1], Alison Shaw[2], Kirstie L Haywood[3] and Esther Crawley[1].

1. Centre for Child and Adolescent Health, Bristol Medical School, University of Bristol, Barley House, Oakfield Grove, Bristol, BS8 2BN. Email: roxanne.parslow@bristol.ac.uk Telephone: 0117 331 0180. (§ Corresponding Author)
2. Centre for Primary Care Research, Bristol Medical School, University of Bristol, Canynge Hall, Bristol, BS8 2PS.
3. Warwick Research in Nursing, Division of Health Sciences, Warwick Medical School, University of Warwick, Health Sciences, Room A108, RCN Research Institute, University of Warwick, Coventry, CV4 7AL.

Confidential: For Review Only

Key Words

Chronic Fatigue Syndrome/ Myalgic Encephalopathy (CFS/ME), adolescents, fatigue, qualitative.

Word Count

Abstract: 291; Text: 3228

Tables: 1; References: 40

What is known about this subject

- Paediatric CFS/ME is relatively common peaking in adolescents between 10–19 years of age and disrupts an important time in their development.
- Fatigue and payback are defining features of the condition, however, a range of aspects of fatigue are currently described in adults with little consensus.
- There are no studies describing adolescent's experiences of fatigue and payback in detail.

What this study adds

- All adolescents describe unique aspects of fatigue in CFS/ME, how it fluctuates naturally day to day but can also get worse following activity (payback).
- The individual experience of fatigue varies in severity, frequency, the amount of activity taken to cause payback as well as the resulting impact on function.
- The variation in the experience of fatigue needs to be taken into account in treatment and when measuring outcomes in the condition.

Confidential: For Review Only

ABSTRACT

Objective

As part of a larger qualitative study to explore the impact of paediatric CFS/ME and what improvement in fatigue and disability are important, interviews were undertaken with adolescents and their parents. This paper focuses on their descriptions of fatigue, fluctuation and payback.

Design and setting

Semi structured qualitative interviews were undertaken between December 2014 and February 2015. Adolescents and parents were interviewed separately. Participants were recruited from a single specialist paediatric chronic fatigue service. Interviews were audio recorded, transcribed verbatim and analysed using thematic analysis.

Participants

We interviewed 21 adolescents and their parents (20 mothers and 2 fathers). The adolescents were aged between 12-17 years of age (mean age 14.4 years), mild to moderately affected by CFS/ME (not housebound) and the majority were female (16/21).

Results

All adolescents with CFS/ME reported fatigue, a natural fluctuation of the condition, with good days and bad days as well as an increase in symptoms after activity (payback). However, adolescents varied in their descriptions of fatigue, symptoms, and the associated impact on their daily lives. The variations included: fatigue versus a collection of symptoms, constant versus variable symptom frequency, and variable

Confidential: For Review Only

symptom severity. There were differences between participants in the amount of activity taken to cause payback. The impact of fatigue and symptoms on function ranged from: limiting the duration and amount of leisure activities, struggling with daily activities (e.g. self-care) to no activity (sedentary).

Conclusions

Fatigue, fluctuation of the condition, and payback after activity are described by all adolescents with CFS/ME in this study. However, the individual experience in terms of how they describe it and the degree and impact varies. The variation in the experience of fatigue needs to be taken into account in treatment and when measuring outcomes in the condition.

Confidential: For Review Only

BMJ Paediatrics Open

INTRODUCTION

Chronic Fatigue Syndrome/ Myalgic Encephalopathy (CFS/ME) is characterised by severe disabling fatigue and can include diverse physical and cognitive symptoms [1,2]. Paediatric CFS/ME is relatively common [3-6], with the incidence reported to peak in adolescents (10–19 years of age) [7]. CFS/ME disrupts the lives of young people; some can become bedbound [8], miss school [9,1011] and stop seeing friends [12-14].

Fatigue is the defining feature of CFS/ME and 'post-exertional malaise' or 'payback' ("physically drained after mild activity") [15] is a universal symptom [16] and key to discriminating CFS/ME patients from other patient populations [17]. However, CFS/ME is heterogeneous; in a recent study, adult CFS/ME patients were found to have one of 6 symptom-based profiles (phenotypes) [16]. The concept of 'fatigue' is complex as a range of aspects are currently described and measured in CFS/ME and there is little agreement on which are most important [18]. Consensus is needed in order to be able to accurately assess the impact of fatigue on patients and measure improvement from treatment. Recently, adults with CFS/ME described the nature of fatigue as three distinct domains: tiredness, fatigue and exhaustion [19]. There is little qualitative evidence in children and this study aimed to explore the impact of CFS/ME on adolescents and their experiences of fatigue, fluctuation and payback.

Confidential: For Review Only

METHODS

Study design

As part of a larger qualitative study exploring the impact of paediatric CFS/ME, semi structured interviews were undertaken with adolescents with CFS/ME and their parents (separately). In four interviews, a parent was present in the room when their child was interviewed. A section of the topic guide incorporated a card ranking exercise. Participants were asked to rank 15 different areas of life impacted by having CFS/ME identified through previous work [13,20], in order of importance, and discuss each card in further detail. The draft topic guide was reviewed by a secondary school, Young Person's Advisory Group (YPAG) to ensure it was age appropriate.

Participants

Adolescents aged between 12- 17 years, diagnosed with mild to moderate CFS/ME (not housebound) [2] and their parents, were recruited from a specialist paediatric chronic fatigue service in South West England. We aimed to recruit a range of participants (age, gender, and disease severity) through maximum variation purposive sampling [21].

Data collection and analysis

Families were approached in outpatient clinics, given the appropriate patient information sheets and later contacted by a researcher to arrange an interview. Written consent was obtained from both parents and adolescents in person, prior to the start of the interview. Interviews were undertaken in participant's own homes and one in the hospital. Interviews continued until we reached data saturation where new interviews produced little or no change to the coding [22].

Confidential: For Review Only

Interviews were recorded on an encrypted digital audio recorder and transcribed verbatim. Transcripts were checked for accuracy, anonymised and uploaded to NVIVO 10 [23] to help organise the data and undertake thematic analysis to report patterns (themes) within data [24]. An initial read through gave an overview of recurring issues of importance to participants. The data was then coded line by line to develop a long list of codes using participants' own language. As interviews progressed, new codes were added to reflect the differences in the data (e.g. mild versus moderate severity). The coding was then reviewed and merged or separated to create broader higher level themes [25]. Finally, a summary of the findings with a focus on fatigue, fluctuation and payback was produced.

An interim analysis (of the first three interviews coded by RP, NA, DB & EC) was used to refine questions and pursue emerging themes [26]. Overall, the coding was found to be consistent. Some participants talked about 'changes over the course of the illness' therefore this theme was pursued in subsequent interviews. Ten transcripts (5 adolescents, 5 parents) were double coded (NA) and compared to improve the trustworthiness of the analyses [26].

Confidential: For Review Only

RESULTS

Participants

We interviewed 43 participants: 21 adolescents (12-17 years old, mean 14.4 years), and their parents (20 mothers and 2 fathers). The majority of adolescents were female (16/21) and mildly affected by CFS/ME (17/21) [2]. Five families initially approached did not participate due to lack of time or being too ill at the time.

Summary of findings

All adolescents with CFS/ME report fatigue, a natural fluctuation of the condition, as well as an increase in fatigue and symptoms after activity (payback). However, the experience was highly variable between participants. The results describe: 1) Adolescents descriptions of fatigue (fatigue vs collection of symptoms, variable symptom frequency and severity); 2) fluctuation of fatigue and symptoms (good days and bad days); 3) increase in fatigue and symptoms following activity (payback) and 4) the impact of on function (limiting leisure activities, reduced daily activities and becoming sedentary).

Adolescents descriptions of fatigue

All adolescents reported tiredness and a lack of energy. Adolescents mostly used the term “tired”, but also described “drowsiness” feeling “worn out”, “heavy” or “drained”. Problems sleeping and difficulties waking up were key features associated with fatigue, "waking up is the hardest bit” (C10, female 13).

Confidential: For Review Only

"I would wake up and I would just feel like I'm dead, like just like I can't get up out of bed. It's horrible" (C17, male 17)

Parents confirmed that mornings were problematic:

"…it was a struggle to get her up big, big struggle to get her up. We would be in and out of her bedroom for ages trying to wake her up, or keep her awake" (P19, mother of female 15).

Fatigue versus a collection of symptoms

Some adolescents described only experiencing "tiredness", whereas others described a collection of severe symptoms:

"There's no really symptoms of it for me, there's no pain or anything, apart from tiredness" (C16, male, 12)

"…it's just when it's a combination of being tired, headache, feeling sick, like achy, then it's just like exhausting." (C7, female 17)

Some adolescents felt tiredness was the most bothersome symptom, whereas for others, symptoms such as headaches and feeling sick had the most significant impact:

"Yeah, I'd be able to live with my headaches…It's really just exhaustion they stop, it stops you doing things" (C1, female 14)

"Probably getting rid of the headaches and the sickness, because the tiredness isn't too bad, but constantly feeling sick is really annoying." (C11, female 16)

Confidential: For Review Only

Constant versus variable symptom frequency

How often symptoms occurred differed between participants, as well as changing on a daily basis. Some adolescents described how certain symptoms were constant, "Achiness, headaches are the main two that they are constant" (C7, female 17). Tiredness was mostly constant "tired all the time" (C5, male 12). Parents reflected on this too "he was just so tired all the time" (P16, mother of male 12). However, for some adolescents it varied, "sometimes I'm tired, sometimes I'm not" (C16, male 12).

Variable symptom severity

Adolescents and their parents often described different severities of symptoms that were changeable. Adolescents described symptoms as being: "really bad", "really worn out". For some, other symptoms became worse with severe tiredness, "symptoms are like, not as bad as they are when I'm really tired." (C3, female 13). Adolescents could articulate different levels of tiredness and the effect this had on their ability to do things:

> *"…like there can be a tiredness where like you just you don't want to get up, and there can be one where you just like you can't do much stuff. So you can be tired and you can be really tired" (C12, male 13).*

> *"…she calls it her normal tiredness and her bad tiredness." (P9, mother of female 13)*

Confidential: For Review Only

Fluctuation of fatigue and symptoms

Adolescents and parents reflected on how CFS/ME naturally fluctuates. CFS/ME was felt to be "unpredictable". Parents described the variation in their child's symptoms "he might have one minor symptom on a good day." (P12, mother of male 13) and physical function, "one day she could be going out for a walk then the next day she might asleep" (P6, mother of female 15). Adolescents and parents felt that fluctuation occurred independent of a trigger or overexertion the previous day and found the unpredictability bothersome:

"I think it does fluctuate on its own... so yesterday was obviously a bad day, but the day before that I hadn't done anything kind of heavy "(C18, female, 17)

"it's not like we pushed too hard." (P1, mother of female 14).

Good versus bad days

Adolescents and parents recognised patterns of good and bad days:

"I have good days most of the week, and then feel terrible for about three days" (C12, male 13)

"She's not been well, she doesn't really have many good days" (P6, mother of female 15).

Adolescents described bad days where there was an increase in symptoms and tiredness, they found it harder to do things and they got low in mood: "the bad days, you just, you can't er, really er, get out of bed" (C2, female 12). Parents felt they were able to instantly recognise a worse day based on adolescent's physical signs

Confidential: For Review Only

and mood, “All I have to do is look at her, I can just tell in her eyes.” (P1, mother of female 14).

In contrast to bad days, adolescents painted a wholly different picture of how they felt on good days, such as having energy, less severe symptoms, an increase in activities: “I do go to school more when I’m feeling better” (C4, female 13). Adolescents recognised that they felt happier and were more sociable on better days, “I'll be all happy and I'll be downstairs and talking to everyone non-stop…” (C19, female 15) and parents described their child with CFS/ME as having two different personalities which allow them to tell what type of day they are having “when she’s feeling better she’s a different child” (P9 mother of female 13).

Increase in fatigue and symptoms following activity (payback)

As well as the natural fluctuation of the condition, adolescents with CFS/ME and their parents talked about the ‘payback’ that can occur after activity. All adolescents described an extreme level of tiredness and feeling ill with CFS/ME that can occur after activity, “but if I do any type of activity I will be worse the next day” (C3, female 13). Payback was described as: “wiped out”, “absolute crash”, “knocked me out”, “zonked out” and “out cold”.

There were differences in the amount and type of activity taken to result in payback. Some adolescents felt they got payback from all types of activity: “but if I do any type of activity I will be worse the next day” (C3, female, aged 13). Other adolescents got payback from managing several activities in one day:

Confidential: For Review Only

"a full day of college, and then driving lesson, my concentration for that hour it's fine, but then it completely knocks me for like that evening. So I just have to go careful really." (C8, female 17).

Adolescents questioned whether enjoyable activities were worth it and were often very bothered by payback: it impacted their desire to participate as they were aware of the potential consequences and therefore it 'held them back' from doing things.

"I will feel completely wiped out and I won't want to do it again." (C5, male 12).

The impact of fatigue and symptoms on function

Limiting leisure activities (amount and duration)

Adolescents were limited in the amount of time they could spend on activities, which ranged from minutes, "five minutes" to hours, "an hour at the most". One parent commented how her child might be able to do a one off short activity like lift an object but would struggle to sustain it "because that's an instantaneous one off thing and the others are maintained exertion" (P20, mother of female 13). Adolescents described how even if they attempted activity, this would be for a short duration and they would suddenly feel limited:

"…I'd had two lessons and I just felt like I just couldn't carry on, I just felt really heavy…" (C18, female 17)

As a result, participants reduced school attendance, sport, hobbies and leisure activities due to their physical limitations. They wanted to be able to do what normal

Confidential: For Review Only

teenagers their age were doing and not have to think about how long they could do it for.

"…I can't go out for too long otherwise I will be really tired". (C16, male 12)

Struggling with basic daily activities

Some adolescents described severe tiredness: "falling asleep and not being able to keep my eyes open." (C6, female 15). Tiredness directly impacted on adolescents' movement, mobility and physical ability to do daily activities. Those who were moderately affected at the time of the interview more often described problems with self-care such as washing, "I can't like have a bath by myself" (C3, female 13) and getting dressed, "I could hardly even get dressed because I could barely stand up" (C12, male 13) as a direct result of fatigue. Adolescents and parents talked about particular problems with walking. This ranged from being limited in walking up stairs and short distances to not being able to sustain walking for a prolonged period of time:

"…I like limped every time I walked because like my legs would feel really weak" (C12, male 13)

"I still go out for like walks but I don't go as far as when I'm ill" (C13, female 17).

No activity (sedentary)

When fatigue and symptoms were worse, this resulted in adolescents being very sedentary "I would just sit in bed or on the sofa and watch TV for the whole day,

Confidential: For Review Only

because that's all that I could do." (C16, male 12). Parents reflected on how their adolescents would "just sit around" and they often had to physically help their child, "we were carrying her up the stairs". Due to their physical limitations they often did not leave the house:

> *"have to stay at home probably most of the time on the sofa asleep rather than doing anything else."(C11, female 16)*

Adolescents felt like life was "dull" as they were always tired and couldn't enjoy activities: "nothing is exciting or fun anymore." Adolescents also described a lack of motivation to do things and withdrawal "I will just stay upstairs for the day. Don't really want to socialise." (C19, female 15).

Changes over the course of the illness (table 1)

As well as fluctuation of the condition in the present, participants often described changes over time and being more severely affected at the onset of CFS/ME. Improvements were often linked to treatment and naturally learning to manage the condition. Sleep was regularly referred to, often improved through medication. An improvement in fatigue or specific symptoms was described with increased energy that allowed adolescents to carry out activities that were previously limited. Some adolescents recalled pushing themselves previously, leading to payback and fluctuation. However, not all adolescents talked about improvements, some had relapsed.

Confidential: For Review Only

Table 1. Improved areas of life according to adolescents and parents.

Improved Areas	Child Quotes	Parental Quotes
**Sleep**	"Don't really get sleep problems anymore, I'm on amitriptyline." (C12, male 13)	"She has before we started the programme thing she was basically asleep all the time, just asleep, because we didn't know what was wrong with her." (P6, mother of female 17)
**Fatigue/ Tiredness**	"Just even like standing up would be tiring. I don't think I probably talked to anyone during those times, it was just really horrible." (C14, female 15)	"...tiredness as well, he was just so tired all the time." (P16, mother of male 12)<br>"But at one stage we were carrying her up the stairs, she didn't have the energy. It's been quite hard…" (P13, mother of female17)
**Specific symptoms**	"My head would be really bad, like there would be times when I would almost like just fall or where I just need to sit down wherever I was, like immediately." (C12, male 13)	"Yeah, because initially it seemed the tiredness triggered off those very bad headaches didn't it? Or they seemed to come together, and those were what we were battling dreadfully with…" (P14, mother of female 15)<br>"Interviewer: What symptoms did you say had improved? P17: Well he just doesn't get them as often..." (P17, mother of male 15)
**Fluctuation**	"I would say the fluctuations from day to day are a lot less, so I have a lot more good days and a lot less bad days" (C13, female 17).	"And fluctuation was a bigger issue then. This is- when it all started these were... I mean everyday was really difficult, some fluctuation definitely which meant that we couldn't really plan anything." (P19, mother of female 15)
**Payback**	"So anything I did I was literally always getting payback, it didn't matter what I was doing I would still like get it. Now it's not so bad." (C11, female 16)<br>"...payback and crashing because it's quite hard to know, would probably have been slightly higher	"...she would crash for days, because she had over exerted herself. Whereas now she knows she has to pace herself, so we don't get that, we're starting to kind of move on from that…" (P11 mother of female 16)

Confidential: For Review Only

	because especially like when you first get it you try to do stuff and then you just get like thrown back really hard all the time." (C15, female 16)	
**Physical function- daily activities**	"Yeah, really struggled with getting up or just getting motivated, like have to stay at home probably most of the time on the sofa asleep rather than doing anything else." (C11, female 16)	"A year ago I would say daily activities, that was probably the biggest because, you know, you could forget about school altogether, you know, helping your 12 year old get washed and dressed is a bit of a shock to the system" (P16, mother of male 16)
**Activities- hobbies, longer duration**	"Interviewer: So are there any things that are improved since a year ago if you see what I mean? C13: Yeah, I do a lot more of my hobbies, and I go to college a lot more than I did at school." (C13, female 17)<br>"Well I 'm much more energetic, I can do a lot more, I can play on my computer a lot more now, but back when I was really bad I couldn't really go on it at all, and I can do Hama beads and stuff like that now. So it's much better now." (C16, male 12)	"…I think she does a lot more now than she used to…" (P11, mother of female 16)

Confidential: For Review Only

DISCUSSION

Fatigue, fluctuation and payback were described by all adolescents with CFS/ME in this study. However, the individual experience in terms of how they describe it and the degree and impact varies. This study highlights key aspects of fatigue described by adolescents with CFS/ME including: sleep problems, problems waking, fluctuation, payback (feeling tired after activity), problems with the duration of activities, daily activities, mobility and being sedentary.

Strengths and limitations of the study

This is the largest qualitative study of adolescents with CFS/ME and their parents to date (n=43). The sample included adolescents across a wide age range. However, fewer males and moderately affected patients were recruited reducing opportunities to explore differences they might experience [21]. The sample was limited to English speaking families from a single site. Nevertheless, it is the largest specialist service in the U.K covering a wide region. Most adolescents were interviewed alone without a parent present which may have reduced socially desirable answers [27,28]. An interactive card ranking exercise was also incorporated into the interview which allowed adolescents to control the interview process [29]. The data analysis was systematic and rigorous; double coding contributed to the checking and interpretation of data by independent researchers [25,30].

Previous Research

Payback was described by all adolescents and has been found to be a universal symptom in adults with CFS/ME [16]. In a recent study, adults with CFS/ME described fatigue as three distinct domains: tiredness (feeling heavy, sleep disturbance), fatigue (e.g. waking up tired, reduced daily activities) and exhaustion (e.g. physical

Confidential: For Review Only

collapse, ‘so tired I fall over’) [19]. The same aspects were identified in this study. However the term ‘payback’ was used instead of exhaustion. Adolescents described instances where they were very sedentary, unable to continue with activities indicating ‘fatigue’. Differences between mild and moderately affected patients were highlighted as those moderately affected described more problems with self-care.

This study highlights the extremely variable nature of CFS/ME. All experienced good days and bad days appearing better on good days and able to do more, often returning to school or activities. This led to some people questioning their illness. The lack of knowledge and understanding of the fluctuating nature of CFS/ME may explain some of the scepticism surrounding CFS/ME [31-36]. There is a need to educate schools, peers and the wider public in order to support young people living with the condition.

Conclusion

In this study, adolescents described key aspects of fatigue, fluctuation of symptoms, and the impact this had on their activity. Paediatricians need to be aware of how adolescents describe the physical experience of CFS/ME. If patients are able to communicate specific symptoms more effectively, research has shown better adherence to treatment and a better therapeutic alliance [37]. No existing child outcome measure covers these issues[38]. A new condition-specific child outcome measure should include these aspects of CFS/ME in order to detect subtle changes in a patient’s condition over time and demonstrate change from treatment [39,40].

Confidential: For Review Only

Acknowledgments

We would like to thank all the participants who took part in the study. We are grateful for the support of the clinicians at the specialist paediatric CFS/ME service who helped to identify and recruit participants.

Funding

This work was supported by a University of Bristol PhD Scholarship. EC is funded by the National Institute for Health Research (Senior Research Fellowship, SRF-2013-06-013). The views expressed in this publication are those of the authors and not necessarily those of the NHS, the National Institute for Health Research or the Department of Health.

Competing interests

All authors declare they have no financial or non-financial interests that may be relevant to the submitted work. EC was the medical advisor for the Association for Young people with ME (AYME) until 2017.

Authors' contributions

RP, EC, AS and KLH designed the study. RP, NA and DB collected the data. RP and NA analysed the data. All authors contributed to the interpretation of results and to drafting this paper. All authors have read and approved the final version of the manuscript.

Data Sharing

No additional data available.

Ethical Approval

Full ethical approval was obtained from the NRES Committee North West (08/04/2014, ref 14/NW/0170). An amendment to use one-to-one interviews with

Confidential: For Review Only

adolescents, parents and clinicians who are unable to attend focus groups and to include exploration of important outcomes was approved (21/10/2014, ref 14/NW/0770). Relevant R&D approval was obtained from the RNHRD (20/06/2014, ref-RBB 427).

Confidential: For Review Only

REFERENCES

1. RCPCH. *Evidence Based Guideline for the Management of CFS/ME (Chronic Fatigue Syndrome/Myalgic Encephalopathy) in Children and Young People.* London: Royal College of Paediatrics and Child Health;2004.
2. NICE. Chronic fatigue syndrome/myalgic encephalomyelitis (or encephalopathy) Diagnosis and management of CFS/ME in adults and children. In. Vol NICE clinical guideline 53. London: National Institute for Health and Care Excellence; 2007.
3. Rimes KA, Goodman R, Hotopf M, Wessely S, Meltzer H, Chalder T. Incidence, prognosis, and risk factors for fatigue and chronic fatigue syndrome in adolescents: a prospective community study. *Pediatrics.* 2007;119(3):603-609.
4. Chalder T, Goodman R, Wessely S, Hotopf M, Meltzer H. Epidemiology of chronic fatigue syndrome and self reported myalgic encephalomyelitis in 5-15 year olds: cross sectional study. *Br Med J.* 2003;327:654-655.
5. Crawley EM, Emond AM, Sterne JA. Unidentified Chronic Fatigue Syndrome/myalgic encephalomyelitis (CFS/ME) is a major cause of school absence: surveillance outcomes from school-based clinics. *BMJ Open.* 2011;1(2):e000252.
6. Crawley E, Hughes R, Northstone K, Tilling K, Emond A, Sterne JA. Chronic disabling fatigue at age 13 and association with family adversity. *Pediatrics.* 2012;130(1):e71-e79.
7. Bakken I, Tveito K, Gunnes N, et al. Two age peaks in the incidence of chronic fatigue syndrome/myalgic encephalomyelitis: a population-based registry study from Norway. *BMC Med.* 2014;12(1):167.
8. Rangel L, Garralda ME, Levin M, Roberts H. The course of severe chronic fatigue syndrome in childhood. *J R Soc Med.* 2000;93(3):129-134.
9. Winger A, Kvarstein G, Wyller VB, et al. Health related quality of life in adolescents with chronic fatigue syndrome: a cross-sectional study. *Health and quality of life outcomes.* 2015;13:96.
10. Crawley E, Sterne JA. Association between school absence and physical function in paediatric chronic fatigue syndrome/myalgic encephalopathy. *Arch Dis Child.* 2009;94(10):752-756.
11. Crawley EM, Emond AM, Sterne JA. Unidentified Chronic Fatigue Syndrome/myalgic encephalomyelitis (CFS/ME) is a major cause of school absence: surveillance outcomes from school-based clinics. *British medical Journal Open.* 2011;1(2):1-6.
12. Taylor RR, O'Brien J, Kielhofner G, Lee SW, Katz B, Mears C. The occupational and quality of life consequences of chronic fatigue syndrome/myalgic encephalomyelitis in young people. *Br J Occup Ther.* 2010;73(11):524-530.
13. Parslow R, Patel A, Beasant L, Haywood K, Johnson D, Crawley E. What matters to children with CFS/ME? A conceptual model as the first stage in developing a PROM. *Arch Dis Child.* 2015;100(12):1141-1147.
14. Parslow RM, Harris S, Broughton J, et al. Children's experiences of chronic fatigue syndrome/myalgic encephalomyelitis (CFS/ME): a systematic review and meta-ethnography of qualitative studies. *BMJ open.* 2017;7(1).
15. Jason L, Jessen T, Porter N, Boulton A, Gloria-Njoku M. Examining types of fatigue among individuals with ME/CFS. *Disability Studies Quarterly.* 2009;29(3).
16. Collin SM, Nikolaus S, Heron J, Knoop H, White PD, Crawley E. Chronic fatigue syndrome (CFS) symptom-based phenotypes in two clinical cohorts of adult patients in the UK and The Netherlands. *J Psychosom Res.* 2016;81:14-23.
17. Jason LA, Evans M, Brown M, et al. Fatigue scales and chronic fatigue syndrome: Issues of sensitivity and specificity. *Disability studies quarterly: DSQ.* 2011;31(1).
18. Dittner AJ, Wessely SC, Brown RG. The assessment of fatigue. A practical guide for clinicians and researchers. *J Psychosom Res.* 2004;56(2):157-170.

Confidential: For Review Only

19. Olson K, Zimka O, Stein E. The Nature of Fatigue in Chronic Fatigue Syndrome. *Qual Health Res.* 2015;25(10):1410-1422.
20. Parslow RM, Shaw A, Haywood KL, Crawley E. Important factors to consider when treating children with chronic fatigue syndrome/myalgic encephalomyelitis (CFS/ME): perspectives of health professionals from specialist services. *BMC Pediatr.* 2017;17(1):43.
21. Ritchie J, Lewis J, Nicholls CM, Ormston R. *Qualitative research practice: A guide for social science students and researchers. Second Edition.* Sage Publication Ltd; 2014.
22. Guest G. How Many Interviews Are Enough?: An Experiment with Data Saturation and Variability. *Field Methods.* 2006;18(1):59-82.
23. QSR International Pty Ltd. NVivo qualitative data analysis software, Version 10. 2014.
24. Braun V, Clarke V. Using Thematic Analysis in Psychology. *QualResPsych.* 2006;3:77-101.
25. Pope C, Mays N. *Qualitative research in health care.* Blackwell Publishing Ltd; 2006.
26. Pope C, Ziebland S, Mays N. Analysing qualitative data. In: *Qualitative Research in Health Care.* London: BMJ Books; 2000:75-88.
27. Mauthner M. Methodological aspects of collecting data from children: Lessons from three research projects. *Children & Society.* 1997;11(1):16-28.
28. Garth B, Murphy GC, Reddihough DS. Perceptions of participation: Child patients with a disability in the doctor–parent–child partnership. *Patient Educ Couns.* 2009;74(1):45-52.
29. Eder D, Fingerson L. Interviewing children and adolecents. In: Gubrium JF, Holstein JA, eds. *Handbook of interview research: Context and method.* Sage; 2002.
30. Barbour RS. Checklists for improving rigour in qualitative research: a case of the tail wagging the dog? *BMJ: British Medical Journal.* 2001;322(7294):1115.
31. Cooper L. Myalgic Encephalomyelitis and the medical encounter1. *Sociol Health Illn.* 1997;19(2):186-207.
32. Åsbring P, Närvänen A-L. Ideal versus reality: physicians perspectives on patients with chronic fatigue syndrome (CFS) and fibromyalgia. *J Adv Nurs.* 2003;57(4):711-720.
33. Chew-Graham C, Dixon R, Shaw JW, Smyth N, Lovell K, Peters S. Practice Nurses' views of their role in the management of Chronic Fatigue Syndrome/Myalagic Encephalitis: a qualitative study. *BMC Nurs.* 2009;8:2.
34. Chew-Graham C, Dowrick C, Wearden A, Richardson V, Peters S. Making the Diagnosis of Chronic Fatigue Syndrome/Myalgic Encephalitis in Primary Care: A Qualitative Study. *BMC Fam Pract.* 2010;11(16).
35. Raine R, Carter S, Sensky T, Black N. General practitioners' perceptions of chronic fatigue syndrome and beliefs about its management, compared with irritable bowel syndrome: qualitative study. *BMJ.* 2004;328(7452):1354-1357.
36. Bayliss K, Goodall M, Chisholm A, et al. Overcoming the barriers to the diagnosis and management of chronic fatigue syndrome/ME in primary care: a meta synthesis of qualitative studies. *BMC Fam Pract.* 2014;15(44):1-11.
37. Snyder CF, Aaronson NK, Choucair AK, et al. Implementing patient-reported outcomes assessment in clinical practice: a review of the options and considerations. *Qual Life Res.* 2012;21(8):1305-1314.
38. Haywood KL, Collin SM, Crawley E. Assessing severity of illness and outcomes of treatment in children with Chronic Fatigue Syndrome/Myalgic Encephalomyelitis (CFS/ME): a systematic review of patient-reported outcome measures (PROMs). *Child Care Health Dev.* 2014;40(6):806-824.
39. McKenna SP. Measuring patient-reported outcomes: moving beyond misplaced common sense to hard science. *BMC Med.* 2011;9:86.
40. Cano SJ, Hobart JC. The problem with health measurement. *Patient Preference and Adherence.* 2011;5:279-290.

Confidential: For Review Only

BMJ Paediatrics Open

Confidential: For Review Only

Adolescent's descriptions of fatigue, fluctuation and payback in Chronic Fatigue Syndrome/Myalgic Encephalopathy (CFS/ME): Interviews with adolescents and parents

Journal:	*BMJ Paediatrics Open*
Manuscript ID	bmjpo-2018-000281.R1
Article Type:	Original article
Date Submitted by the Author:	20-Apr-2018

Complete List of Authors:	Parslow, Roxanne; University of Bristol, Centre for Child and Adolescent Health<br>Anderson, Nina; University of Bristol, Centre for Child and Adolescent Health<br>Byrne, Danielle; University of Bristol, Centre for Child and Adolescent Health<br>Shaw, Alison; University of Bristol, 2. Centre for Primary Care Research<br>Haywood, Kirstie; University of Warwick, Warwick Research in Nursing<br>Crawley, Esther; University of Bristol School of Social and Community Medicine, Centre for Child and Adolescent Health

Keywords:	Chronic Fatigue Syndrome, Adolescent Health, Qualitative research, Patient perspective

SCHOLARONE™
Manuscripts

BMJ Paediatrics Open

Adolescent's descriptions of fatigue, fluctuation and payback in Chronic Fatigue Syndrome/Myalgic Encephalopathy (CFS/ME): Interviews with adolescents and parents

Roxanne, M. Parslow[1], Nina Anderson[1], Danielle Byrne[1], Alison Shaw[2], Kirstie L Haywood[3] and Esther Crawley[1§].

1. Centre for Child and Adolescent Health, Bristol Medical School, University of Bristol, Barley House, Oakfield Grove, Bristol, BS8 2BN. Email: esther.crawley@bristol.ac.uk Telephone: 0117 331 0180. (§ Corresponding Author)
2. Centre for Primary Care Research, Bristol Medical School, University of Bristol, Canynge Hall, Bristol, BS8 2PS.
3. Warwick Research in Nursing, Division of Health Sciences, Warwick Medical School, University of Warwick, Health Sciences, Room A108, RCN Research Institute, University of Warwick, Coventry, CV4 7AL.

Key Words

Chronic Fatigue Syndrome/ Myalgic Encephalopathy (CFS/ME), adolescents, fatigue, qualitative.

Word Count

Abstract: 293; Text: 3417

Tables: 1; References: 49

Confidential: For Review Only

What is known about this subject

- Paediatric CFS/ME is relatively common peaking in adolescents between 10–19 years of age and disrupts an important time in their development.
- Fatigue and payback are defining features of the condition and these are well described in adults.
- There are no studies describing adolescent's experiences of fatigue and payback in detail.

What this study adds

- All adolescents describe unique aspects of fatigue in CFS/ME; how it fluctuates naturally day to day but can also get worse following activity (payback).
- The individual experience of fatigue varies in severity, frequency, the amount of activity taken to cause payback as well as the resulting impact on function.
- The variation in the experience of fatigue needs to be taken into account in treatment and when measuring outcomes in the condition.

Confidential: For Review Only

ABSTRACT

Objective

As part of a larger qualitative study to explore outcomes important in paediatric CFS/ME and what improvement in fatigue and disability are key, interviews were undertaken with adolescents and their parents. This paper focuses on their descriptions of fatigue, fluctuation of symptoms and payback.

Design and setting

Semi structured qualitative interviews were undertaken between December 2014 and February 2015. Adolescents and parents were interviewed separately. Participants were recruited from a single specialist paediatric chronic fatigue service. Interviews were audio recorded, transcribed verbatim and analysed using thematic analysis.

Participants

We interviewed 21 adolescents and their parents (20 mothers and 2 fathers). The adolescents were aged between 12-17 years of age (mean age 14.4 years), mild to moderately affected by CFS/ME (not housebound) and the majority were female (16/21).

Results

All adolescents with CFS/ME reported fatigue, a natural fluctuation of the condition, with good days and bad days as well as an increase in symptoms after activity (payback). However, adolescents varied in their descriptions of fatigue, symptoms, and the associated impact on their daily lives. The variations included: fatigue versus a collection of symptoms, constant versus variable symptom frequency, and variable

Confidential: For Review Only

symptom severity. There were differences between participants in the amount of activity taken to cause payback. The impact of fatigue and symptoms on function ranged from: limiting the duration and amount of leisure activities, struggling with daily activities (e.g. self-care) to no activity (sedentary).

Conclusions

Fatigue, fluctuation of the condition, and payback after activity are described by all adolescents with CFS/ME in this study. However, the individual experience in terms of how they describe it and the degree and impact varies. The variation in the experience of fatigue needs to be taken into account in treatment and when measuring outcomes in the condition.

Confidential: For Review Only

INTRODUCTION

Chronic Fatigue Syndrome/ Myalgic Encephalopathy (CFS/ME) is characterised by severe disabling fatigue and can include diverse physical and cognitive symptoms [1,2]. Paediatric CFS/ME is relatively common with a prevalence of 0.6-2.4% [3-8], and the incidence reported to peak in adolescents (10–19 years of age) [9]. CFS/ME disrupts the lives of young people; some can become bedbound [10], miss school [11,1213] and stop seeing friends [14-16].

Fatigue is the defining feature of CFS/ME and ‘post-exertional malaise’ or ‘payback’ (“physically drained after mild activity”) [17] is a universal symptom [18] and key to discriminating CFS/ME patients from other patient populations [19]. However, CFS/ME is heterogeneous; in a recent study, adult CFS/ME patients were found to have one of 6 symptom-based profiles (phenotypes) [18]. The concept of ‘fatigue’ and ‘payback’ are complex as a range of aspects are currently described and measured in CFS/ME and there is little agreement on which are most important [20,21]. Consensus is needed in order to be able to accurately assess the impact of fatigue and payback on patients and measure improvement from treatment. Recently, adults with CFS/ME described the nature of fatigue as three distinct domains: tiredness, fatigue and exhaustion [22]. However, there is less qualitative evidence in children. As part of a larger study investigating outcomes important in paediatric CFS/ME, a large amount of data arose relating to fatigue, symptoms and payback. This paper aims to describe adolescent’s experiences of fatigue, fluctuation of symptoms and payback specifically.

Confidential: For Review Only

METHODS

Study design

As part of a larger qualitative study exploring outcomes important in paediatric CFS/ME, semi structured interviews were undertaken with adolescents with CFS/ME and their parents (separately). We felt both perspectives were important to understand the overall impact of CFS/ME. Previous work exploring how quality of life is impacted by a childhood disorder was reviewed to help inform the development of the topic guide [23-26]. The guide included questions on the overall impact of CFS/ME, how participants would know if they were better/ worse or recovered and incorporated a card ranking exercise (Appendix A). Participants were asked to rank 15 different areas of life impacted by having CFS/ME identified through previous work [15,27], in order of importance, and discuss each card in further detail. The draft topic guide was reviewed by a secondary school, Young Person's Advisory Group (YPAG) to ensure it was age appropriate. For this paper, we analysed data collected when participants specifically talked about fatigue, fluctuation and payback.

Participants

Adolescents aged between 12- 17 years, diagnosed with mild to moderate CFS/ME (not housebound) [2] and their parents, were recruited from a specialist paediatric chronic fatigue service in South West England. We aimed to recruit a range of participants (age, gender, and disease severity) through maximum variation purposive sampling [28].

Data collection and analysis

Families were approached in outpatient clinics, given the appropriate patient information sheets and later contacted by a researcher to arrange an interview.

Confidential: For Review Only

Written consent was obtained from both parents and adolescents in person, prior to the start of the interview. Interviews continued until we reached data saturation where new interviews produced little or no change to the coding [29].

Interviews were recorded on an encrypted digital audio recorder and transcribed verbatim. Transcripts were checked for accuracy, anonymised and uploaded to NVIVO 10 [30] to help organise the data and undertake thematic analysis to report patterns (themes) within data [31]. Data analysis started using deductive (anticipated) codes of the health outcomes outlined in the card ranking exercise identified from previous research. Transcripts were then read and re-read to further develop inductive (emergent) codes using participants' own language of their descriptions of fatigue, fluctuation and payback in detail, producing a long list of codes. Transcripts for the subgroups (adolescents vs parents and mild-moderate severity) were coded separately in NVIVO [30] and the groups were then compared to search for similarities and differences in the data [32,33]. The coding was then reviewed and merged or separated to create broader higher level themes [34].

An interim analysis (of the first three interviews coded by RP, NA, DB & EC) was used to refine questions and pursue emerging themes [35]. Overall, the coding was found to be consistent. Some participants talked about 'changes over the course of the illness' therefore this theme was pursued in subsequent interviews. Ten transcripts (5 adolescents, 5 parents) were double coded (NA) and compared to improve the trustworthiness of the analyses [35].

Confidential: For Review Only

RESULTS

Participants

We interviewed 43 participants: 21 adolescents (12-17 years old, mean 14.4 years), and their parents (20 mothers and 2 fathers- two parents from one family were interviewed). The majority of adolescents were female (16/21) and mildly affected by CFS/ME (17/21) [2]. Adolescents were mostly interviewed alone, however, in four interviews, a parent was present in the room when their child was interviewed. Interviews were undertaken in participant's own homes and one in the hospital. Five families initially approached did not participate due to lack of time or being too ill at the time.

Summary of findings

All adolescents with CFS/ME report fatigue, a natural fluctuation of the condition, as well as an increase in fatigue and symptoms after activity (payback). However, the experience was highly variable between participants. The results describe: 1) Adolescents descriptions of fatigue (fatigue vs collection of symptoms, variable symptom frequency and severity); 2) fluctuation of fatigue and symptoms (good days and bad days); 3) increase in fatigue and symptoms following activity (payback) and 4) the impact of fatigue on function (limiting leisure activities, reduced daily activities and becoming sedentary).

Confidential: For Review Only

Adolescents descriptions of fatigue

All adolescents reported tiredness and a lack of energy. Adolescents mostly used the term “tired”, but also described “drowsiness” feeling “worn out”, “heavy” or “drained”. Problems sleeping and difficulties waking up were key features associated with fatigue, "waking up is the hardest bit” (C10, female 13).

> *“I would wake up and I would just feel like I’m dead, like just like I can’t get up out of bed. It’s horrible” (C17, male 17)*

Parents confirmed that mornings were problematic:

> *“…it was a struggle to get her up big, big struggle to get her up. We would be in and out of her bedroom for ages trying to wake her up, or keep her awake” (P19, mother of female 15).*

Fatigue versus a collection of symptoms

Some adolescents described only experiencing “tiredness”, whereas others described a collection of severe symptoms:

> *“There’s no really symptoms of it for me, there’s no pain or anything, apart from tiredness” (C16, male, 12)*

> *“…it’s just when it’s a combination of being tired, headache, feeling sick, like achy, then it’s just like exhausting.” (C7, female 17)*

Some adolescents felt tiredness was the most bothersome symptom, whereas for others, symptoms such as headaches and feeling sick had the most significant impact:

Confidential: For Review Only

"Yeah, I'd be able to live with my headaches…It's really just exhaustion they stop, it stops you doing things" (C1, female 14)

"Probably getting rid of the headaches and the sickness, because the tiredness isn't too bad, but constantly feeling sick is really annoying." (C11, female 16)

Constant versus variable symptom frequency

How often symptoms occurred differed between participants, as well as changing on a daily basis. Some adolescents described how certain symptoms were constant, "Achiness, headaches are the main two that they are constant" (C7, female 17). Tiredness was mostly constant, "tired all the time" (C5, male 12). Parents reflected on this too, "he was just so tired all the time" (P16, mother of male 12). However, for some adolescents it varied, "sometimes I'm tired, sometimes I'm not" (C16, male 12).

Variable symptom severity

Adolescents and their parents often described how some symptoms would change in how severe they were Adolescents described symptoms as being: "really bad", "really worn out". For some, other symptoms became worse with severe tiredness, "symptoms are like, not as bad as they are when I'm really tired." (C3, female 13). Adolescents could articulate different levels of tiredness and the effect this had on their ability to do things:

"…like there can be a tiredness where like you just you don't want to get up, and there can be one where you just like you can't do much stuff. So you can be tired and you can be really tired" (C12, male 13).

Confidential: For Review Only

"…she calls it her normal tiredness and her bad tiredness." (P9, mother of female 13)

Fluctuation of fatigue and symptoms

Adolescents and parents reflected on how CFS/ME naturally fluctuates. CFS/ME was felt to be "unpredictable". Parents described the variation in their child's symptoms "he might have one minor symptom on a good day." (P12, mother of male 13) and physical function, "one day she could be going out for a walk then the next day she might asleep" (P6, mother of female 15). Adolescents and parents felt that fluctuation occurred independent of a trigger or overexertion the previous day and found the unpredictability bothersome:

"I think it does fluctuate on its own... so yesterday was obviously a bad day, but the day before that I hadn't done anything kind of heavy "(C18, female, 17)

"it's not like we pushed too hard." (P1, mother of female 14).

Good versus bad days

Adolescents and parents recognised patterns of good and bad days:

"I have good days most of the week, and then feel terrible for about three days" (C12, male 13)

"She's not been well, she doesn't really have many good days" (P6, mother of female 15).

Adolescents described bad days where there was an increase in symptoms and tiredness, they found it harder to do things and they got low in mood: "the bad days, you just, you can't er, really er, get out of bed" (C2, female 12). Parents felt they

Confidential: For Review Only

were able to instantly recognise a worse day based on adolescent's physical signs and mood, "All I have to do is look at her, I can just tell in her eyes." (P1, mother of female 14).

In contrast to bad days, adolescents painted a wholly different picture of how they felt on good days, such as having energy, less severe symptoms, an increase in activities: "I do go to school more when I'm feeling better" (C4, female 13). Adolescents recognised that they felt happier and were more sociable on better days, "I'll be all happy and I'll be downstairs and talking to everyone non-stop…" (C19, female 15) and parents described their child with CFS/ME as having two different personalities which allow them to tell what type of day they are having "when she's feeling better she's a different child" (P9 mother of female 13).

Increase in fatigue and symptoms following activity (payback)

As well as the natural fluctuation of the condition, adolescents with CFS/ME and their parents talked about the 'payback' that can occur after activity. All adolescents described an extreme level of tiredness and feeling ill with CFS/ME that can occur after activity, "but if I do any type of activity I will be worse the next day" (C3, female 13). Payback was described as: "wiped out", "absolute crash", "knocked me out", "zonked out" and "out cold".

There were differences in the amount and type of activity taken to result in payback. Some adolescents felt they got payback from all types of activity: "but if I do any type of activity I will be worse the next day" (C3, female, aged 13). Other adolescents got payback from managing several activities in one day:

Confidential: For Review Only

> *"a full day of college, and then driving lesson, my concentration for that hour it's fine, but then it completely knocks me for like that evening. So I just have to go careful really." (C8, female 17).*

Adolescents questioned whether enjoyable activities were worth it and were often very bothered by payback; it impacted their desire to participate as they were aware of the potential consequences and therefore, it 'held them back' from doing things.

> *"I will feel completely wiped out and I won't want to do it again." (C5, male 12).*

The impact of fatigue and symptoms on function

Limiting leisure activities

Adolescents were limited in the amount of time they could spend on activities, which ranged from minutes, "five minutes" to hours, "an hour at the most". One parent commented how her child might be able to do a one off short activity, like 'lift an object' but would struggle to sustain it "because that's an instantaneous one off thing and the others are maintained exertion" (P20, mother of female 13). Adolescents described how even if they attempted activity, this would be for a short duration and they would suddenly feel limited:

> *"…I'd had two lessons and I just felt like I just couldn't carry on, I just felt really heavy…" (C18, female 17)*

As a result, participants reduced school attendance, sport, hobbies and leisure activities due to their physical limitations. They wanted to be able to do what normal

Confidential: For Review Only

teenagers their age were doing and not have to think about how long they could do it for.

"…I can't go out for too long otherwise I will be really tired". (C16, male 12)

Struggling with basic daily activities

Some adolescents described severe tiredness: "falling asleep and not being able to keep my eyes open." (C6, female 15). Tiredness directly impacted on adolescents' movement, mobility and physical ability to do daily activities. Those who were moderately affected at the time of the interview more often described problems with self-care such as washing, "I can't like have a bath by myself" (C3, female 13) and getting dressed, "I could hardly even get dressed because I could barely stand up" (C12, male 13) as a direct result of fatigue. Adolescents and parents talked about particular problems with walking. This ranged from being limited in walking up stairs and short distances, to not being able to sustain walking for a prolonged period of time:

"…I like limped every time I walked because like my legs would feel really weak" (C12, male 13)

"I still go out for like walks but I don't go as far as when I'm ill" (C13, female 17).

No activity (sedentary)

When fatigue and symptoms were worse, this resulted in adolescents being very sedentary, "I would just sit in bed or on the sofa and watch TV for the whole day,

Confidential: For Review Only

because that's all that I could do." (C16, male 12). Parents reflected on how their child would: "just sit around" and they often had to physically help their child, "we were carrying her up the stairs". Due to their physical limitations, adolescents often did not leave the house:

> *"have to stay at home probably most of the time on the sofa asleep rather than doing anything else."(C11, female 16)*

Adolescents felt like life was "dull" as they were always tired and couldn't enjoy activities: "nothing is exciting or fun anymore." They also described a lack of motivation to do things and withdrawal: "I will just stay upstairs for the day. Don't really want to socialise." (C19, female 15).

Changes over the course of the illness (table 1)

In addition to the "natural fluctuation" and "increase in symptoms after payback", participants often described changes over time. Improvements were often linked to treatment and naturally learning to manage the condition. Sleep was regularly referred to, often improved through medication. An improvement in fatigue or specific symptoms was described with increased energy that allowed adolescents to carry out activities that were previously limited. Some adolescents recalled pushing themselves previously, leading to payback and fluctuation. However, not all adolescents talked about improvements, some had relapsed.

Confidential: For Review Only

Table 1. Changes over time according to adolescents and parents.

Improved Areas	Child Quotes	Parental Quotes
**Sleep**	"Don't really get sleep problems anymore, I'm on amitriptyline." (C12, male 13)	"She has before we started the programme thing she was basically asleep all the time, just asleep, because we didn't know what was wrong with her." (P6, mother of female 17)
**Fatigue/ Tiredness**	"Just even like standing up would be tiring. I don't think I probably talked to anyone during those times, it was just really horrible." (C14, female 15)	"...tiredness as well, he was just so tired all the time." (P16, mother of male 12)<br>"But at one stage we were carrying her up the stairs, she didn't have the energy. It's been quite hard…" (P13, mother of female17)
**Specific symptoms**	"My head would be really bad, like there would be times when I would almost like just fall or where I just need to sit down wherever I was, like immediately." (C12, male 13)	"Yeah, because initially it seemed the tiredness triggered off those very bad headaches didn't it? Or they seemed to come together, and those were what we were battling dreadfully with…" (P14, mother of female 15)<br>"Interviewer: What symptoms did you say had improved? P17: Well he just doesn't get them as often..." (P17, mother of male 15)
**Fluctuation**	"I would say the fluctuations from day to day are a lot less, so I have a lot more good days and a lot less bad days" (C13, female 17).	"And fluctuation was a bigger issue then. This is- when it all started these were... I mean everyday was really difficult, some fluctuation definitely which meant that we couldn't really plan anything." (P19, mother of female 15)
**Payback**	"So anything I did I was literally always getting payback, it didn't matter what I was doing I would still like get it. Now it's not so bad." (C11, female 16)<br>"...payback and crashing because it's quite hard to know, would probably have been slightly higher	"...she would crash for days, because she had over exerted herself. Whereas now she knows she has to pace herself, so we don't get that, we're starting to kind of move on from that…" (P11 mother of female 16)

Confidential: For Review Only

	because especially like when you first get it you try to do stuff and then you just get like thrown back really hard all the time." (C15, female 16)	
**Physical function- daily activities**	"Yeah, really struggled with getting up or just getting motivated, like have to stay at home probably most of the time on the sofa asleep rather than doing anything else." (C11, female 16)	"A year ago I would say daily activities, that was probably the biggest because, you know, you could forget about school altogether, you know, helping your 12 year old get washed and dressed is a bit of a shock to the system" (P16, mother of male 16)
**Activities- hobbies, longer duration**	"Interviewer: So are there any things that are improved since a year ago if you see what I mean? C13: Yeah, I do a lot more of my hobbies, and I go to college a lot more than I did at school." (C13, female 17)<br>"Well I 'm much more energetic, I can do a lot more, I can play on my computer a lot more now, but back when I was really bad I couldn't really go on it at all, and I can do Hama beads and stuff like that now. So it's much better now." (C16, male 12)	"…I think she does a lot more now than she used to…" (P11, mother of female 16)

Confidential: For Review Only

DISCUSSION

Fatigue, fluctuation and payback were described by all adolescents with CFS/ME in this study. However, the individual experience in terms of how they describe it and the degree and impact varies. This study highlights key aspects of fatigue described by adolescents with CFS/ME including: sleep problems, problems waking, fluctuation, payback (feeling tired after activity), problems with the duration of activities, daily activities, mobility and being sedentary.

Strengths and limitations of the study

This is the largest qualitative study of adolescents with CFS/ME and their parents to date (n=43). The sample included adolescents across what may be considered a wide developmental age range. However, fewer males and moderately affected patients were recruited reducing opportunities to explore differences they might experience [28]. Only two fathers were interviewed as mothers were mostly present when recruiting families from outpatient clinics. Further research is needed to explore their experiences. The sample was limited to English speaking families from a single site. Nevertheless, it is the largest specialist service in the U.K covering a wide region. Most adolescents were interviewed alone without a parent present which may have reduced socially desirable answers [36,37]. Differences between sub groups were considered in the analysis; no major differences emerged between children and parents when describing fatigue, symptoms and their impact, however, moderately affected children described more problems with self-care. An interactive card ranking exercise was also incorporated into the interview which allowed adolescents to control the interview process [38]. The data analysis was systematic

Confidential: For Review Only

and rigorous; double coding contributed to the checking and interpretation of data by independent researchers [34,39].

Previous Research

Payback was described by all adolescents and has been found to be a universal symptom in adults with CFS/ME [18]. In a recent study, adults with CFS/ME described fatigue as three distinct domains: tiredness (feeling heavy, sleep disturbance), fatigue (e.g. waking up tired, reduced daily activities) and exhaustion (e.g. physical collapse, 'so tired I fall over') [22]. The same aspects were identified in this study. However the term 'payback' was used instead of exhaustion. Adolescents described instances where they were very sedentary, unable to continue with activities indicating 'fatigue'. Differences between mild and moderately affected patients were highlighted as those moderately affected described more problems with self-care.

This study highlights the extremely variable nature of CFS/ME. All experienced good days and bad days appearing better on good days and able to do more, often returning to school or activities. This led to some people questioning their illness. The lack of knowledge and understanding of the fluctuating nature of CFS/ME may explain some of the scepticism surrounding CFS/ME [40-45]. There is a need to educate schools, peers and the wider public in order to support young people living with the condition.

Conclusion

In this study, adolescents described key aspects of fatigue, fluctuation of symptoms, and the impact this had on their activity. Paediatricians need to be aware of how adolescents describe the physical experience of CFS/ME. If patients are able to communicate specific symptoms more effectively, research has shown better

Confidential: For Review Only

adherence to treatment and a better therapeutic alliance [46]. No existing child outcome measure covers these issues[47]. A new condition-specific child outcome measure should include these aspects of CFS/ME in order to detect subtle changes in a patient's condition over time and demonstrate change from treatment [48,49].

Confidential: For Review Only

Acknowledgments

We would like to thank all the participants who took part in the study. We are grateful for the support of the clinicians at the specialist paediatric CFS/ME service who helped to identify and recruit participants.

Funding

This work was supported by a University of Bristol PhD Scholarship. EC is funded by the National Institute for Health Research (Senior Research Fellowship, SRF-2013-06-013). The views expressed in this publication are those of the authors and not necessarily those of the NHS, the National Institute for Health Research or the Department of Health.

Competing interests

All authors declare they have no financial or non-financial interests that may be relevant to the submitted work. EC was the medical advisor for the Association for Young people with ME (AYME) until 2017.

Authors' contributions

RP, EC, AS and KLH designed the study. RP, NA and DB collected the data. RP and NA analysed the data. All authors contributed to the interpretation of results and to drafting this paper. All authors have read and approved the final version of the manuscript.

Data Sharing

No additional data available.

Ethical Approval

Full ethical approval was obtained from the NRES Committee North West (08/04/2014, ref 14/NW/0170). An amendment to use one-to-one interviews with

Confidential: For Review Only

adolescents, parents and clinicians who are unable to attend focus groups and to include exploration of important outcomes was approved (21/10/2014, ref 14/NW/0770). Relevant R&D approval was obtained from the RNHRD (20/06/2014, ref-RBB 427).

Confidential: For Review Only

REFERENCES

1. RCPCH. *Evidence Based Guideline for the Management of CFS/ME (Chronic Fatigue Syndrome/Myalgic Encephalopathy) in Children and Young People.* London: Royal College of Paediatrics and Child Health;2004.
2. NICE. Chronic fatigue syndrome/myalgic encephalomyelitis (or encephalopathy) Diagnosis and management of CFS/ME in adults and children. In. Vol NICE clinical guideline 53. London: National Institute for Health and Care Excellence; 2007.
3. Rimes KA, Goodman R, Hotopf M, Wessely S, Meltzer H, Chalder T. Incidence, prognosis, and risk factors for fatigue and chronic fatigue syndrome in adolescents: a prospective community study. *Pediatrics.* 2007;119(3):e603-609.
4. Chalder T, Goodman R, Wessely S, Hotopf M, Meltzer H. Epidemiology of chronic fatigue syndrome and self reported myalgic encephalomyelitis in 5-15 year olds: cross sectional study. *Bmj.* 2003;327(7416):654-655.
5. Crawley EM, Emond AM, Sterne JA. Unidentified Chronic Fatigue Syndrome/myalgic encephalomyelitis (CFS/ME) is a major cause of school absence: surveillance outcomes from school-based clinics. *BMJ Open.* 2011;1(2):e000252.
6. Crawley E ea. Chronic disabling fatigue at age 13 and association with family adversity. - PubMed - NCBI. 2012.
7. Haines LC, Saidi G, Cooke RW. Prevalence of severe fatigue in primary care. *Arch Dis Child.* 2005;90(4):367-368.
8. Nijhof SL, Maijer K, Bleijenberg G, Uiterwaal CS, Kimpen JL, van de Putte EM. Adolescent chronic fatigue syndrome: prevalence, incidence, and morbidity. *Pediatrics.* 2011;127(5):e1169-1175.
9. Bakken I, Tveito K, Gunnes N, et al. Two age peaks in the incidence of chronic fatigue syndrome/myalgic encephalomyelitis: a population-based registry study from Norway. *BMC Med.* 2014;12(1):167.
10. Rangel L, Garralda ME, Levin M, Roberts H. The course of severe chronic fatigue syndrome in childhood. *J R Soc Med.* 2000;93(3):129-134.
11. Winger A, Kvarstein G, Wyller VB, et al. Health related quality of life in adolescents with chronic fatigue syndrome: a cross-sectional study. *Health and quality of life outcomes.* 2015;13:96.
12. Crawley E, Sterne JA. Association between school absence and physical function in paediatric chronic fatigue syndrome/myalgic encephalopathy. *Arch Dis Child.* 2009;94(10):752-756.
13. Crawley EM, Emond AM, Sterne JA. Unidentified Chronic Fatigue Syndrome/myalgic encephalomyelitis (CFS/ME) is a major cause of school absence: surveillance outcomes from school-based clinics. *British medical Journal Open.* 2011;1(2):1-6.
14. Taylor RR, O'Brien J, Kielhofner G, Lee SW, Katz B, Mears C. The occupational and quality of life consequences of chronic fatigue syndrome/myalgic encephalomyelitis in young people. *Br J Occup Ther.* 2010;73(11):524-530.
15. Parslow R, Patel A, Beasant L, Haywood K, Johnson D, Crawley E. What matters to children with CFS/ME? A conceptual model as the first stage in developing a PROM. *Arch Dis Child.* 2015;100(12):1141-1147.
16. Parslow RM, Harris S, Broughton J, et al. Children's experiences of chronic fatigue syndrome/myalgic encephalomyelitis (CFS/ME): a systematic review and meta-ethnography of qualitative studies. *BMJ open.* 2017;7(1).
17. Jason L, Jessen T, Porter N, Boulton A, Gloria-Njoku M. Examining types of fatigue among individuals with ME/CFS. *Disability Studies Quarterly.* 2009;29(3).
18. Collin SM, Nikolaus S, Heron J, Knoop H, White PD, Crawley E. Chronic fatigue syndrome (CFS) symptom-based phenotypes in two clinical cohorts of adult patients in the UK and The Netherlands. *J Psychosom Res.* 2016;81:14-23.

Confidential: For Review Only

19. Jason LA, Evans M, Brown M, et al. Fatigue scales and chronic fatigue syndrome: Issues of sensitivity and specificity. *Disability studies quarterly: DSQ.* 2011;31(1).
20. Dittner AJ, Wessely SC, Brown RG. The assessment of fatigue. A practical guide for clinicians and researchers. *J Psychosom Res.* 2004;56(2):157-170.
21. Jason LA, Evans M, So S, Scott J, Brown A. Problems in Defining Post-Exertional Malaise. *Journal of Prevention & Intervention in the Community.* 2015;43(1):20-31.
22. Olson K, Zimka O, Stein E. The Nature of Fatigue in Chronic Fatigue Syndrome. *Qual Health Res.* 2015;25(10):1410-1422.
23. Ronen GM, Rosenbaum P, Law M, Streiner DL. Health-Related Quality of Life in Childhood Disorders: A Modified Focus Group Technique to Involve Children. *Qual Life Res.* 2001;10:71-79.
24. McEwan MJ, Espie CA, Metcalfe J, Brodie MJ, Wilson MT. Quality of life and psychosocial development in adolescents with epilepsy: a qualitative investigation using focus group methods. *Seizure.* 2004;13(1):15-31.
25. Morris C, Liabo K, Wright P, Fitzpatrick R. Development of the Oxford ankle foot questionnaire: finding out how children are affected by foot and ankle problems. *Child Care Health Dev.* 2007;33(5):559-568.
26. Rahi JS, Tadic V, Keeley S, Lewando-Hundt G. Capturing children and young people's perspectives to identify the content for a novel vision-related quality of life instrument. *Ophthalmology.* 2011;118(5):819-824.
27. Parslow RM, Shaw A, Haywood KL, Crawley E. Important factors to consider when treating children with chronic fatigue syndrome/myalgic encephalomyelitis (CFS/ME): perspectives of health professionals from specialist services. *BMC Pediatr.* 2017;17(1):43.
28. Ritchie J, Lewis J, Nicholls CM, Ormston R. *Qualitative research practice: A guide for social science students and researchers. Second Edition.* Sage Publication Ltd; 2014.
29. Guest G. How Many Interviews Are Enough?: An Experiment with Data Saturation and Variability. *Field Methods.* 2006;18(1):59-82.
30. QSR International Pty Ltd. NVivo qualitative data analysis software, Version 10. 2014.
31. Braun V, Clarke V. Using Thematic Analysis in Psychology. *QualResPsych.* 2006;3:77-101.
32. Boeije H. A purposeful approach to the constant comparative method in the analysis of qualitative interviews. . *Quality and quantity.* 2002;36(4):391-409.
33. Corbin J, Strauss A. Grounded Theory Research - Procedures, Canons And Evaluative Criteria. *Zeitschrift Fur Soziologie.* 1990;19(6):418-427.
34. Pope C, Mays N. *Qualitative research in health care.* Blackwell Publishing Ltd; 2006.
35. Pope C, Ziebland S, Mays N. Analysing qualitative data. In: *Qualitative Research in Health Care.* London: BMJ Books; 2000:75-88.
36. Mauthner M. Methodological aspects of collecting data from children: Lessons from three research projects. *Children & Society.* 1997;11(1):16-28.
37. Garth B, Murphy GC, Reddihough DS. Perceptions of participation: Child patients with a disability in the doctor–parent–child partnership. *Patient Educ Couns.* 2009;74(1):45-52.
38. Eder D, Fingerson L. Interviewing children and adolecents. In: Gubrium JF, Holstein JA, eds. *Handbook of interview research: Context and method.* Sage; 2002.
39. Barbour RS. Checklists for improving rigour in qualitative research: a case of the tail wagging the dog? *BMJ: British Medical Journal.* 2001;322(7294):1115.
40. Cooper L. Myalgic Encephalomyelitis and the medical encounter1. *Sociol Health Illn.* 1997;19(2):186-207.
41. Åsbring P, Närvänen A-L. Ideal versus reality: physicians perspectives on patients with chronic fatigue syndrome (CFS) and fibromyalgia. *J Adv Nurs.* 2003;57(4):711-720.
42. Chew-Graham C, Dixon R, Shaw JW, Smyth N, Lovell K, Peters S. Practice Nurses' views of their role in the management of Chronic Fatigue Syndrome/Myalagic Encephalitis: a qualitative study. *BMC Nurs.* 2009;8:2.

Confidential: For Review Only

43. Chew-Graham C, Dowrick C, Wearden A, Richardson V, Peters S. Making the Diagnosis of Chronic Fatigue Syndrome/Myalgic Encephalitis in Primary Care: A Qualitative Study. *BMC Fam Pract.* 2010;11(16).
44. Raine R, Carter S, Sensky T, Black N. General practitioners' perceptions of chronic fatigue syndrome and beliefs about its management, compared with irritable bowel syndrome: qualitative study. *BMJ.* 2004;328(7452):1354-1357.
45. Bayliss K, Goodall M, Chisholm A, et al. Overcoming the barriers to the diagnosis and management of chronic fatigue syndrome/ME in primary care: a meta synthesis of qualitative studies. *BMC Fam Pract.* 2014;15(44):1-11.
46. Snyder CF, Aaronson NK, Choucair AK, et al. Implementing patient-reported outcomes assessment in clinical practice: a review of the options and considerations. *Qual Life Res.* 2012;21(8):1305-1314.
47. Haywood KL, Collin SM, Crawley E. Assessing severity of illness and outcomes of treatment in children with Chronic Fatigue Syndrome/Myalgic Encephalomyelitis (CFS/ME): a systematic review of patient-reported outcome measures (PROMs). *Child Care Health Dev.* 2014;40(6):806-824.
48. McKenna SP. Measuring patient-reported outcomes: moving beyond misplaced common sense to hard science. *BMC Med.* 2011;9:86.
49. Cano SJ, Hobart JC. The problem with health measurement. *Patient Preference and Adherence.* 2011;5:279-290.

Confidential: For Review Only

APPENDIX A: Outcomes in Paediatric CFS/ME

Extract from Young Person Topic Guide

Introduction

1. How does having CFS/ME affect your life?

Prompts: Can you tell me more about X, what is it about X, can you describe that…

Recovery

2. How do you know when you are feeling worse?

3. How do you know when you are feeling better?

4. What things if any stay the same whether you are better or worse?

5. How would you know if you were completely better again?

Prompts: What would need to improve for you to say that you are better, by how much?

What would you like to do that you are unable to now?

What things can you cope with if they don't improve/ return to normal?

Outcome Domains

Activity: Present YP with outcome domain cards. Add any domains to the blank cards the YP may have spontaneously brought up. Get YP to select the areas most affected by CFS/ME and rank them in order of importance as to what is most important to improve.

Top 5 domains- explore dimensions of domains (frequency/ severity/ duration/ satisfaction)

Prompts e.g. what symptoms bother you most, what is important about school.

[Take a photo of order].

6. Pick out the top issues/areas of your life most affected by CFS/ME (upto 10).

7. Imagine you were able to improve these areas, rank these in order of what you feel is most important to improve, put the areas at the top you would most like to improve.

Tiredness

Symptoms (pain, headaches, feeling sick, brain fog)

Sleep problems

Daily activities (getting up, getting dressed, going out)

Payback & crashing (tired after activity)

Fluctuation (changing symptoms- good day vs. bad day)

School (attendance, concentrating, keeping up with work)

Activities & hobbies (sports, clubs)

Spending time with friends

Family activities

Confidential: For Review Only

V1.4 _28/01/2015

Mood (feeling down, worrying)

How you feel about yourself (confidence, personality)

Your future (GCSEs, college, jobs)

Independence (doing things without your parents)

Seeing your boyfriend/girlfriend

8. Why have you ranked them in that order?

Top 5 domains- explore dimensions of domains (frequency/ severity/ duration/ satisfaction)

- Prompts e.g. what symptoms bother you most, what is important about school.

9. How might your answer have been different a year ago? [Pick the cards].

- Why were they important?
- In what ways has it changed/ improved?

<u>Ending question</u>

10. Is there anything else about having CFS/ME that you feel is important to you?

Confidential: For Review Only

BMJ Paediatrics Open

Confidential: For Review Only

Adolescent's descriptions of fatigue, fluctuation and payback in Chronic Fatigue Syndrome/Myalgic Encephalopathy (CFS/ME): Interviews with adolescents and parents

Journal:	*BMJ Paediatrics Open*
Manuscript ID	bmjpo-2018-000281.R2
Article Type:	Original article
Date Submitted by the Author:	01-Sep-2018

Complete List of Authors:	Parslow, Roxanne; University of Bristol, Centre for Child and Adolescent Health<br>Anderson, Nina; University of Bristol, Centre for Child and Adolescent Health<br>Byrne, Danielle; University of Bristol, Centre for Child and Adolescent Health<br>Shaw, Alison; University of Bristol, 2. Centre for Primary Care Research<br>Haywood, Kirstie; University of Warwick, Warwick Research in Nursing<br>Crawley, Esther; University of Bristol School of Social and Community Medicine, Centre for Child and Adolescent Health

Keywords:	Chronic Fatigue Syndrome, Adolescent Health, Qualitative research, Patient perspective

SCHOLARONE™ Manuscripts

Adolescent's descriptions of fatigue, fluctuation and payback in Chronic Fatigue Syndrome/Myalgic Encephalopathy (CFS/ME): Interviews with adolescents and parents

Confidential: For Review Only

Roxanne, M. Parslow[1], Nina Anderson[1], Danielle Byrne[1], Alison Shaw[2], Kirstie L Haywood[3] and Esther Crawley[1§].

1. Centre for Child and Adolescent Health, Bristol Medical School, University of Bristol, 1-5 Whiteladies Road, Bristol, BS8 1NU. Email: esther.crawley@bristol.ac.uk Telephone: 0117 331 0180. (§ Corresponding Author)
2. Centre for Primary Care Research, Bristol Medical School, University of Bristol, Canynge Hall, Bristol, BS8 2PS.
3. Warwick Research in Nursing, Division of Health Sciences, Warwick Medical School, University of Warwick, Health Sciences, Room A108, RCN Research Institute, University of Warwick, Coventry, CV4 7AL.

Key Words

Chronic Fatigue Syndrome/ Myalgic Encephalopathy (CFS/ME), adolescents, fatigue, qualitative.

Word Count

Abstract: 268; Text: 2915

References: 40

What is known about this subject

- Paediatric CFS/ME is relatively common peaking in adolescents between 10–19 years of age and disrupts an important time in their development.
- Fatigue and payback are defining features of the condition and these are well described in adults.
- There are no studies describing adolescent's experiences of fatigue and payback in detail.

What this study adds

- All adolescents describe unique aspects of fatigue in CFS/ME; how it fluctuates naturally day to day but can also get worse following activity (payback).
- The individual experience of fatigue varies in severity, frequency, the amount of activity taken to cause payback as well as the resulting impact on function.
- The variation in the experience of fatigue needs to be taken into account in treatment.

Confidential: For Review Only

ABSTRACT

Objective

As part of a larger qualitative study to explore outcomes important in paediatric CFS/ME and what improvements in fatigue and disability are key, interviews were undertaken with adolescents and their parents. This paper focuses on their descriptions of fatigue, fluctuation of symptoms and payback.

Design and setting

Semi structured qualitative interviews were undertaken between December 2014 and February 2015. Adolescents and parents were interviewed separately. Participants were recruited from a single specialist paediatric chronic fatigue service. Interviews were audio recorded, transcribed verbatim and analysed using thematic analysis.

Participants

We interviewed 21 adolescents and their parents (20 mothers and 2 fathers). The adolescents were aged between 12-17 years of age (mean age 14.4 years), mild to moderately affected by CFS/ME (not housebound) and the majority were female (16/21).

Results

All adolescents with CFS/ME reported fatigue, a natural fluctuation of the condition, with good days and bad days as well as an increase in symptoms after activity (payback). However, adolescent's descriptions of fatigue, symptoms, and the associated impact on their daily lives differed. The variations included: fatigue versus a collection of symptoms, constant versus variable symptoms, and variable symptom

Confidential: For Review Only

severity. There were differences between participants in the amount of activity taken to cause payback. The impact of fatigue and symptoms on function ranged from: limiting the duration and amount of leisure activities, struggling with daily activities (e.g. self-care) to no activity (sedentary).

Conclusions

Fatigue, fluctuation of the condition, and payback after activity are described by all adolescents with CFS/ME in this study. However, the individual experience in terms of how they describe it and the degree and impact varies.

Confidential: For Review Only

INTRODUCTION

Chronic Fatigue Syndrome/ Myalgic Encephalopathy (CFS/ME) is characterised by severe disabling fatigue and can include diverse physical and cognitive symptoms [1,2]. Paediatric CFS/ME is relatively common with a prevalence of 0.6-2.4% [3-8], and the incidence reported to peak in adolescents (10–19 years of age) [9]. CFS/ME disrupts the lives of young people; some can become bedbound [10], miss school [11,1213] and stop seeing friends [14-16].

Fatigue is the defining feature of CFS/ME and 'post-exertional malaise' or 'payback' ("physically drained after mild activity") [17] is a universal symptom [18] and key to discriminating CFS/ME patients from other patient populations [19]. However, CFS/ME is heterogeneous; in a recent study, adult CFS/ME patients were found to have one of 6 symptom-based profiles (phenotypes) [18]. The concepts of 'fatigue' and 'payback' are complex as a range of aspects are currently described and measured in CFS/ME and there is little agreement on which are most important [20,21]. Consensus is needed in order to be able to accurately assess the impact of fatigue and payback on patients and measure improvement from treatment. Recently, adults with CFS/ME described the nature of fatigue as three distinct domains: tiredness, fatigue and exhaustion [22]. However, there is less qualitative evidence in children. As part of a larger study investigating outcomes important in paediatric CFS/ME[23], detailed data arose relating to fatigue, fluctuation of symptoms and payback and this paper aims to describe adolescents' experiences and descriptions of these domains.

Confidential: For Review Only

METHODS

Study design

As part of a larger qualitative study exploring outcomes important in paediatric CFS/ME, semi structured interviews were undertaken with adolescents with CFS/ME and their parents (separately) between December 2014 and February 2015. We felt both perspectives were important to understand the overall impact of CFS/ME.

Participants

Adolescents aged between 12- 17 years, diagnosed with mild to moderate CFS/ME (not housebound) [2] and their parents, were recruited from a specialist paediatric chronic fatigue service in South West England. We aimed to recruit a range of participants (age, gender, and disease severity) through maximum variation purposive sampling [24]. Families were approached in outpatient clinics, given the appropriate patient information sheets and later contacted by a researcher to arrange an interview. Written consent was obtained from both parents and adolescents in person, prior to the start of the interview.

Data collection

Previous work exploring how quality of life is impacted by a childhood disorder was reviewed to help inform the development of the topic guide [25-28]. The guide included questions on the overall impact of CFS/ME, how participants would know if they were better/ worse or recovered and incorporated a card ranking exercise (Appendix A). Participants were asked to rank 15 different areas of life impacted by having CFS/ME identified through previous work [15,29], in order of importance, and discuss each card in further detail. Fatigue, fluctuation and payback were previously identified as important to children with CFS/ME [15,29] and therefore included in the

Confidential: For Review Only

card ranking exercise. This paper analyses the detailed qualitative data collected in these areas. Adolescents were mostly interviewed alone, however, in four interviews, a parent was present in the room when their child was interviewed. Interviews were undertaken in participant's own homes and one in the hospital.

Data analysis

Interviews were recorded on an encrypted digital audio recorder and transcribed verbatim. Transcripts were checked for accuracy, anonymised and uploaded to NVIVO 10 [30] to help organise the data and undertake thematic analysis to report patterns (themes) within data [31]. Transcripts for the subgroups (adolescents vs parents and mild-moderate severity) were coded separately in NVIVO and the groups were then compared to search for similarities and differences in the data [32,33].

Data analysis started using deductive (anticipated) codes of the health outcomes outlined in the card ranking exercise identified from previous research. Transcripts were then read and re-read to further develop inductive (emergent) codes using participants' own language of their descriptions of fatigue, symptoms, fluctuation and payback in detail, producing a long list of codes (Appendix B). The coding was reviewed as interviews progressed merging or separating codes based on participants' descriptions[34]. Interviews continued until we reached data saturation where new interviews produced little or no change to the inductive codes [35]. Ten transcripts (5 adolescents, 5 parents) were double coded (NA) and compared to improve the trustworthiness of the analyses [36].

Confidential: For Review Only

RESULTS

Participants

We interviewed 43 participants: 21 adolescents (12-17 years old, mean 14.4 years), and their parents (20 mothers and 2 fathers- two parents from one family were interviewed). The majority of adolescents were female (16/21) and mildly affected by CFS/ME (17/21) [2]. Five families initially approached did not participate due to lack of time or being too ill at the time.

Summary of findings

All adolescents with CFS/ME report fatigue, a natural fluctuation of the condition, as well as an increase in fatigue and symptoms after activity (payback). However, the experience was highly variable between participants. The results describe: 1) Adolescents descriptions of fatigue (fatigue vs collection of symptoms, constant vs variable symptoms and variable symptom severity); 2) fluctuation of fatigue and symptoms (good days and bad days); 3) increase in fatigue and symptoms following activity (payback) and 4) the impact of fatigue and symptoms on function (limiting leisure activities, reduced daily activities and becoming sedentary).

Adolescents descriptions of fatigue

All adolescents reported tiredness and a lack of energy. Adolescents mostly used the term “tired”, but also described “drowsiness” feeling “worn out”, “heavy” or “drained”. Problems sleeping and difficulties waking up were key features associated with fatigue, "waking up is the hardest bit” (C10, female 13).

Confidential: For Review Only

> *"I would wake up and I would just feel like I'm dead, like just like I can't get up out of bed. It's horrible" (C17, male 17)*

Parents confirmed that mornings were problematic:

> *"…it was a struggle to get her up big, big struggle to get her up. We would be in and out of her bedroom for ages trying to wake her up, or keep her awake" (P19, mother of female 15).*

Confidential: For Review Only

Fatigue versus a collection of symptoms

Some adolescents described only experiencing "tiredness", whereas others described a collection of severe symptoms:

> *"There's no really symptoms of it for me, there's no pain or anything, apart from tiredness" (C16, male, 12)*

> *"…it's just when it's a combination of being tired, headache, feeling sick, like achy, then it's just like exhausting." (C7, female 17)*

Some adolescents felt tiredness was the most bothersome symptom, whereas for others, symptoms such as headaches and feeling sick had the most significant impact:

> *"Yeah, I'd be able to live with my headaches…It's really just exhaustion they stop, it stops you doing things" (C1, female 14)*

> *"Probably getting rid of the headaches and the sickness, because the tiredness isn't too bad, but constantly feeling sick is really annoying." (C11, female 16)*

Constant versus variable symptoms

How often symptoms occurred differed between participants, as well as changing on a daily basis. Some adolescents described how certain symptoms were constant, "Achiness, headaches are the main two that they are constant" (C7, female 17). Tiredness was mostly constant, "tired all the time" (C5, male 12). Parents reflected on this too, "he was just so tired all the time" (P16, mother of male 12). However, for some adolescents it varied, "sometimes I'm tired, sometimes I'm not" (C16, male 12).

Variable symptom severity

Adolescents and their parents often talked about how some symptoms would change in how severe they were. Symptoms were described as being: "really bad", "really worn out". For some, all symptoms became worse with severe tiredness, "symptoms are like, not as bad as they are when I'm really tired." (C3, female 13). Adolescents could articulate different levels of tiredness and the effect this had on their ability to do things:

> *"…like there can be a tiredness where like you just you don't want to get up, and there can be one where you just like you can't do much stuff. So you can be tired and you can be really tired" (C12, male 13).*

> *"…she calls it her normal tiredness and her bad tiredness." (P9, mother of female 13)*

Confidential: For Review Only

Fluctuation of fatigue and symptoms

Adolescents and parents reflected on how CFS/ME naturally fluctuates. CFS/ME was felt to be "unpredictable". Parents described the variation in their child's symptoms "he might have one minor symptom on a good day." (P12, mother of male 13) and physical function, "one day she could be going out for a walk then the next day she might asleep" (P6, mother of female 15). Adolescents and parents felt that fluctuation occurred independent of a trigger or overexertion the previous day and found the unpredictability bothersome:

"I think it does fluctuate on its own... so yesterday was obviously a bad day, but the day before that I hadn't done anything kind of heavy "(C18, female, 17)

"it's not like we pushed too hard." (P1, mother of female 14).

Good versus bad days

Adolescents and parents recognised patterns of good and bad days:

"I have good days most of the week, and then feel terrible for about three days" (C12, male 13)

"She's not been well, she doesn't really have many good days" (P6, mother of female 15).

Adolescents described bad days where there was an increase in symptoms and tiredness, they found it harder to do things and they got low in mood: "the bad days, you just, you can't er, really er, get out of bed" (C2, female 12). Parents felt they were able to instantly recognise a worse day based on adolescent's physical signs

Confidential: For Review Only

and mood, “All I have to do is look at her, I can just tell in her eyes.” (P1, mother of female 14).

In contrast to bad days, adolescents painted a wholly different picture of how they felt on good days, such as having energy, less severe symptoms, an increase in activities: “I do go to school more when I’m feeling better” (C4, female 13). Adolescents recognised that they felt happier and were more sociable on better days, “I'll be all happy and I'll be downstairs and talking to everyone non-stop…” (C19, female 15) and parents described their child with CFS/ME as having two different personalities which allow them to tell what type of day they are having “when she’s feeling better she’s a different child” (P9 mother of female 13).

Increase in fatigue and symptoms following activity (payback)

As well as the natural fluctuation of the condition, adolescents with CFS/ME and their parents talked about the ‘payback’ that can occur after activity. All adolescents described an extreme level of tiredness and feeling ill with CFS/ME that can occur after activity, “but if I do any type of activity I will be worse the next day” (C3, female 13). Payback was described as: “wiped out”, “absolute crash”, “knocked me out”, “zonked out” and “out cold”.

There were differences in the amount and type of activity taken to result in payback. Some adolescents felt they got payback from all types of activity: “but if I do any type of activity I will be worse the next day” (C3, female, aged 13). Other adolescents got payback from managing several activities in one day:

Confidential: For Review Only

"a full day of college, and then driving lesson, my concentration for that hour it's fine, but then it completely knocks me for like that evening. So I just have to go careful really." (C8, female 17).

Adolescents questioned whether enjoyable activities were worth it and were often very bothered by payback; it impacted their desire to participate as they were aware of the potential consequences and therefore, it 'held them back' from doing things.

"I will feel completely wiped out and I won't want to do it again." (C5, male 12).

The impact of fatigue and symptoms on function

Limiting leisure activities

Adolescents were limited in the amount of time they could spend on activities, which ranged from minutes, "five minutes" to hours, "an hour at the most". One parent commented how her child might be able to do a one off short activity, like 'lift an object' but would struggle to sustain it "because that's an instantaneous one off thing and the others are maintained exertion" (P20, mother of female 13). Adolescents described how even if they attempted activity, this would be for a short duration and they would suddenly feel limited:

"…I'd had two lessons and I just felt like I just couldn't carry on, I just felt really heavy…" (C18, female 17)

As a result, participants reduced school attendance, sport, hobbies and leisure activities due to their physical limitations. They wanted to be able to do what normal

Confidential: For Review Only

teenagers their age were doing and not have to think about how long they could do it for.

"…I can't go out for too long otherwise I will be really tired". (C16, male 12)

Struggling with basic daily activities

Some adolescents described severe tiredness: "falling asleep and not being able to keep my eyes open." (C6, female 15). Tiredness directly impacted on adolescents' movement, mobility and physical ability to do daily activities. Those who were moderately affected at the time of the interview more often described problems with self-care such as washing, "I can't like have a bath by myself" (C3, female 13) and getting dressed, "I could hardly even get dressed because I could barely stand up" (C12, male 13) as a direct result of fatigue. Adolescents and parents talked about particular problems with walking. This ranged from being limited in walking up stairs and short distances, to not being able to sustain walking for a prolonged period of time:

"…I like limped every time I walked because like my legs would feel really weak" (C12, male 13)

"I still go out for like walks but I don't go as far as when I'm ill" (C13, female 17).

No activity (sedentary)

When fatigue and symptoms were worse, this resulted in adolescents being very sedentary, "I would just sit in bed or on the sofa and watch TV for the whole day,

Confidential: For Review Only

because that's all that I could do." (C16, male 12). Parents reflected on how their child would: "just sit around" and they often had to physically help their child, "we were carrying her up the stairs". Due to their physical limitations, adolescents often did not leave the house:

> *"have to stay at home probably most of the time on the sofa asleep rather than doing anything else."(C11, female 16)*

Adolescents felt like life was "dull" as they were always tired and couldn't enjoy activities: "nothing is exciting or fun anymore." They also described a lack of motivation to do things and withdrawal: "I will just stay upstairs for the day. Don't really want to socialise." (C19, female 15).

Confidential: For Review Only

DISCUSSION

Fatigue, fluctuation and payback were described by all adolescents with CFS/ME in this study. However, the individual experience in terms of how they describe it and the degree and impact varies. This study highlights key aspects of fatigue described by adolescents with CFS/ME including: sleep problems, problems waking, fluctuation, payback (feeling tired after activity), problems with the duration of activities, daily activities, mobility and being sedentary.

Strengths and limitations of the study

This is the largest qualitative study of adolescents with CFS/ME and their parents to date (n=43). The sample included adolescents across what may be considered a wide developmental age range. However, fewer males and moderately affected patients were recruited reducing opportunities to explore differences they might experience [24]. Only two fathers were interviewed as mothers were mostly present when recruiting families from outpatient clinics. Further research is needed to explore their experiences. The sample was limited to English speaking families from a single site. Nevertheless, it is the largest specialist service in the U.K covering a wide region. Most adolescents were interviewed alone without a parent present which may have reduced socially desirable answers [37,38]. Differences between sub groups were considered in the analysis; no major differences emerged between children and parents when describing fatigue, symptoms and their impact, however, moderately affected children described more problems with self-care. The data analysis was systematic and rigorous; double coding contributed to the checking and interpretation of data by independent researchers [34,39].

Confidential: For Review Only

Previous Research

Payback was described by all adolescents and has been found to be a universal symptom in adults with CFS/ME [18]. Similar to adults with CFS/ME who described fatigue as: tiredness (feeling heavy, sleep disturbance), fatigue (e.g. waking up tired, reduced daily activities) and exhaustion (e.g. physical collapse, ‘so tired I fall over’) [22], adolescents in this study described the same domains but ‘payback’ was used instead of ‘exhaustion’ and they didn’t always say “fatigue” caused sedentary days. Our results are consistent with previous research where children described the intensity of symptoms fluctuating as well as “overextension” making it worse, resulting in “paying the price” [15,40]

This study details the extremely individual and variable nature of CFS/ME. Whereas one teenager may experience constant fatigue and get payback after managing several activities, another may have a range of symptoms that vary and any activity would result in an increase in symptom severity. All experienced good days and bad days appearing better on good days and able to do more, often returning to school or activities. Differences between mild and moderately affected patients were highlighted as those moderately affected described more problems with self-care.

Conclusion

In this study, adolescents described key aspects of fatigue, fluctuation of symptoms, and the impact this had on their activity. Paediatricians need to be aware of how adolescents describe the physical experience of CFS/ME.

Confidential: For Review Only

Acknowledgments

We would like to thank all the participants who took part in the study. We are grateful for the support of the clinicians at the specialist paediatric CFS/ME service who helped to identify and recruit participants.

Funding

This work was supported by a University of Bristol PhD Scholarship. EC is funded by the National Institute for Health Research (Senior Research Fellowship, SRF-2013-06-013). The views expressed in this publication are those of the authors and not necessarily those of the NHS, the National Institute for Health Research or the Department of Health.

Competing interests

All authors declare they have no financial or non-financial interests that may be relevant to the submitted work. EC was the medical advisor for the Association for Young people with ME (AYME) until 2017.

Authors' contributions

RP, EC, AS and KLH designed the study. RP, NA and DB collected the data. RP and NA analysed the data. All authors contributed to the interpretation of results and to drafting this paper. All authors have read and approved the final version of the manuscript.

Data Sharing

No additional data available.

Ethical Approval

Full ethical approval was obtained from the NRES Committee North West (08/04/2014, ref 14/NW/0170). An amendment to use one-to-one interviews with

Confidential: For Review Only

adolescents, parents and clinicians who are unable to attend focus groups and to include exploration of important outcomes was approved (21/10/2014, ref 14/NW/0770). Relevant R&D approval was obtained from the RNHRD (20/06/2014, ref-RBB 427).

Confidential: For Review Only

REFERENCES

1. RCPCH. *Evidence Based Guideline for the Management of CFS/ME (Chronic Fatigue Syndrome/Myalgic Encephalopathy) in Children and Young People.* London: Royal College of Paediatrics and Child Health;2004.
2. NICE. Chronic fatigue syndrome/myalgic encephalomyelitis (or encephalopathy) Diagnosis and management of CFS/ME in adults and children. In. Vol NICE clinical guideline 53. London: National Institute for Health and Care Excellence; 2007.
3. Rimes KA, Goodman R, Hotopf M, Wessely S, Meltzer H, Chalder T. Incidence, prognosis, and risk factors for fatigue and chronic fatigue syndrome in adolescents: a prospective community study. *Pediatrics.* 2007;119(3):e603-609.
4. Chalder T, Goodman R, Wessely S, Hotopf M, Meltzer H. Epidemiology of chronic fatigue syndrome and self reported myalgic encephalomyelitis in 5-15 year olds: cross sectional study. *Bmj.* 2003;327(7416):654-655.
5. Crawley EM, Emond AM, Sterne JA. Unidentified Chronic Fatigue Syndrome/myalgic encephalomyelitis (CFS/ME) is a major cause of school absence: surveillance outcomes from school-based clinics. *BMJ Open.* 2011;1(2):e000252.
6. Crawley E ea. Chronic disabling fatigue at age 13 and association with family adversity. - PubMed - NCBI. 2012.
7. Haines LC, Saidi G, Cooke RW. Prevalence of severe fatigue in primary care. *Arch Dis Child.* 2005;90(4):367-368.
8. Nijhof SL, Maijer K, Bleijenberg G, Uiterwaal CS, Kimpen JL, van de Putte EM. Adolescent chronic fatigue syndrome: prevalence, incidence, and morbidity. *Pediatrics.* 2011;127(5):e1169-1175.
9. Bakken I, Tveito K, Gunnes N, et al. Two age peaks in the incidence of chronic fatigue syndrome/myalgic encephalomyelitis: a population-based registry study from Norway. *BMC Med.* 2014;12(1):167.
10. Rangel L, Garralda ME, Levin M, Roberts H. The course of severe chronic fatigue syndrome in childhood. *J R Soc Med.* 2000;93(3):129-134.
11. Winger A, Kvarstein G, Wyller VB, et al. Health related quality of life in adolescents with chronic fatigue syndrome: a cross-sectional study. *Health and quality of life outcomes.* 2015;13:96.
12. Crawley E, Sterne JA. Association between school absence and physical function in paediatric chronic fatigue syndrome/myalgic encephalopathy. *Arch Dis Child.* 2009;94(10):752-756.
13. Crawley EM, Emond AM, Sterne JA. Unidentified Chronic Fatigue Syndrome/myalgic encephalomyelitis (CFS/ME) is a major cause of school absence: surveillance outcomes from school-based clinics. *British medical Journal Open.* 2011;1(2):1-6.
14. Taylor RR, O'Brien J, Kielhofner G, Lee SW, Katz B, Mears C. The occupational and quality of life consequences of chronic fatigue syndrome/myalgic encephalomyelitis in young people. *Br J Occup Ther.* 2010;73(11):524-530.
15. Parslow R, Patel A, Beasant L, Haywood K, Johnson D, Crawley E. What matters to children with CFS/ME? A conceptual model as the first stage in developing a PROM. *Arch Dis Child.* 2015;100(12):1141-1147.
16. Parslow RM, Harris S, Broughton J, et al. Children's experiences of chronic fatigue syndrome/myalgic encephalomyelitis (CFS/ME): a systematic review and meta-ethnography of qualitative studies. *BMJ open.* 2017;7(1).
17. Jason L, Jessen T, Porter N, Boulton A, Gloria-Njoku M. Examining types of fatigue among individuals with ME/CFS. *Disability Studies Quarterly.* 2009;29(3).
18. Collin SM, Nikolaus S, Heron J, Knoop H, White PD, Crawley E. Chronic fatigue syndrome (CFS) symptom-based phenotypes in two clinical cohorts of adult patients in the UK and The Netherlands. *J Psychosom Res.* 2016;81:14-23.

Confidential: For Review Only

19. Jason LA, Evans M, Brown M, et al. Fatigue scales and chronic fatigue syndrome: Issues of sensitivity and specificity. *Disability studies quarterly: DSQ.* 2011;31(1).
20. Dittner AJ, Wessely SC, Brown RG. The assessment of fatigue. A practical guide for clinicians and researchers. *J Psychosom Res.* 2004;56(2):157-170.
21. Jason LA, Evans M, So S, Scott J, Brown A. Problems in Defining Post-Exertional Malaise. *Journal of Prevention & Intervention in the Community.* 2015;43(1):20-31.
22. Olson K, Zimka O, Stein E. The Nature of Fatigue in Chronic Fatigue Syndrome. *Qual Health Res.* 2015;25(10):1410-1422.
23. Parslow RM. *Developing a Patient Reported Outcome Measure (PROM) for Children with Chronic Fatigue Syndrome/Myalgic Encephalomyelitis (CFS/ME)* [PhD]. Bristol: Centre for Child and Adolescent Health, Univesity of Bristol; 2016.
24. Ritchie J, Lewis J, Nicholls CM, Ormston R. *Qualitative research practice: A guide for social science students and researchers. Second Edition.* Sage Publication Ltd; 2014.
25. Ronen GM, Rosenbaum P, Law M, Streiner DL. Health-Related Quality of Life in Childhood Disorders: A Modified Focus Group Technique to Involve Children. *Qual Life Res.* 2001;10:71-79.
26. McEwan MJ, Espie CA, Metcalfe J, Brodie MJ, Wilson MT. Quality of life and psychosocial development in adolescents with epilepsy: a qualitative investigation using focus group methods. *Seizure.* 2004;13(1):15-31.
27. Morris C, Liabo K, Wright P, Fitzpatrick R. Development of the Oxford ankle foot questionnaire: finding out how children are affected by foot and ankle problems. *Child Care Health Dev.* 2007;33(5):559-568.
28. Rahi JS, Tadic V, Keeley S, Lewando-Hundt G. Capturing children and young people's perspectives to identify the content for a novel vision-related quality of life instrument. *Ophthalmology.* 2011;118(5):819-824.
29. Parslow RM, Shaw A, Haywood KL, Crawley E. Important factors to consider when treating children with chronic fatigue syndrome/myalgic encephalomyelitis (CFS/ME): perspectives of health professionals from specialist services. *BMC Pediatr.* 2017;17(1):43.
30. QSR International Pty Ltd. NVivo qualitative data analysis software, Version 10. 2014.
31. Braun V, Clarke V. Using Thematic Analysis in Psychology. *QualResPsych.* 2006;3:77-101.
32. Boeije H. A purposeful approach to the constant comparative method in the analysis of qualitative interviews. . *Quality and quantity.* 2002;36(4):391-409.
33. Corbin J, Strauss A. Grounded Theory Research - Procedures, Canons And Evaluative Criteria. *Zeitschrift Fur Soziologie.* 1990;19(6):418-427.
34. Pope C, Mays N. *Qualitative research in health care.* Blackwell Publishing Ltd; 2006.
35. Guest G. How Many Interviews Are Enough?: An Experiment with Data Saturation and Variability. *Field Methods.* 2006;18(1):59-82.
36. Pope C, Ziebland S, Mays N. Analysing qualitative data. In: *Qualitative Research in Health Care.* London: BMJ Books; 2000:75-88.
37. Mauthner M. Methodological aspects of collecting data from children: Lessons from three research projects. *Children & Society.* 1997;11(1):16-28.
38. Garth B, Murphy GC, Reddihough DS. Perceptions of participation: Child patients with a disability in the doctor–parent–child partnership. *Patient Educ Couns.* 2009;74(1):45-52.
39. Barbour RS. Checklists for improving rigour in qualitative research: a case of the tail wagging the dog? *BMJ: British Medical Journal.* 2001;322(7294):1115.
40. Hareide L, Finset A, Wyller VB. Chronic fatigue syndrome: a qualitative investigation of young patient's beliefs and coping strategies. *Disabil Rehabil.* 2011;33(23-24):2255-2263.

Confidential: For Review Only

APPENDIX A: Outcomes in Paediatric CFS/ME

Extract from Young Person Topic Guide

Introduction

1. **How does having CFS/ME affect your life?**

Prompts: Can you tell me more about X, what is it about X, can you describe that…

Recovery

2. **How do you know when you are feeling worse?**
3. **How do you know when you are feeling better?**
4. **What things if any stay the same whether you are better or worse?**

5. **How would you know if you were completely better again?**

Prompts: What would need to improve for you to say that you are better, by how much?
What would you like to do that you are unable to now?
What things can you cope with if they don't improve/ return to normal?

Outcome Domains

Activity: Present YP with outcome domain cards. Add any domains to the blank cards the YP may have spontaneously brought up. Get YP to select the areas most affected by CFS/ME and rank them in order of importance as to what is most important to improve.
Top 5 domains- explore dimensions of domains (frequency/ severity/ duration/ satisfaction)
Prompts e.g. what symptoms bother you most, what is important about school.
[Take a photo of order].

6. **Pick out the top issues/areas of your life most affected by CFS/ME (upto 10).**
7. **Imagine you were able to improve these areas, rank these in order of what you feel is most important to improve, put the areas at the top you would most like to improve.**

Tiredness
Symptoms (pain, headaches, feeling sick, brain fog)
Sleep problems
Daily activities (getting up, getting dressed, going out)
Payback & crashing (tired after activity)
Fluctuation (changing symptoms- good day vs. bad day)
School (attendance, concentrating, keeping up with work)
Activities & hobbies (sports, clubs)
Spending time with friends
Family activities

Confidential: For Review Only

Mood (feeling down, worrying)

How you feel about yourself (confidence, personality)

Your future (GCSEs, college, jobs)

Independence (doing things without your parents)

Seeing your boyfriend/girlfriend

8. Why have you ranked them in that order?

Top 5 domains- explore dimensions of domains (frequency/ severity/ duration/ satisfaction)

- Prompts e.g. what symptoms bother you most, what is important about school.

9. How might your answer have been different a year ago? [Pick the cards].

- Why were they important?
- In what ways has it changed/ improved?

<u>Ending question</u>

10. Is there anything else about having CFS/ME that you feel is important to you?

Confidential: For Review Only

APPENDIX B: Thematic Framework

Overarching themes (deductive)<br>*From card ranking exercise*	Subthemes (inductive)
**FATIGUE AND SYMPTOMS**	**Sleep**<br>Problems at night<br>Sleeping during the day (moderate)<br>**Tiredness and lack of energy**<br>Tired all the time (duration)<br>Feeling quite worn out (severity)<br>**Memory and concentrations**<br>**Pain**<br>Muscle pain and aches<br>Headaches<br>Sore throat<br>Stomach ache<br>Pain quite bad (severity of pain)<br>Sick-nausea-no appetite<br>Dizziness<br>Sensitivity to light<br>Feeling cold<br>Feel horrible, ill<br>Feeling weak<br>**Dimensions of Symptoms**<br>**Only one symptom vs. collection of symptoms**<br>**Frequency of symptoms (all the time, changes)**<br>**Severity of symptoms (its really bad)**
**FLUCTUATION**	**Bad day**<br>Symptoms get worse on bad days<br>Cant do anything<br>More tired<br>Other people notice physical signs-pale<br>Get more payback if do things on a bad day<br>**Good day**<br>Feel more energetic on better days<br>Can do more<br>Feel happier<br>More talkative, sociable<br>No-less symptoms<br>Feel better in the morning-had more sleep<br>Go to school<br>Less tired
**PAYBACK**	Trying to do more tires me out-be completely wiped-cant do anything the next day<br>Have to plan activities<br>Can do things e.g. go into town but get payback<br>Symptoms get worse
**IMPACT ON PHYSICAL FUNCTIONING**	**Limiting leisure activities**<br>Cant go out for too long<br>Cant do what you want to<br>**Problems with daily activities-getting dressed, bathing**<br>Problems walking

Confidential: For Review Only

	Problems climbing stairs<br>Need to use wheelchair<br>**Tiredness-cant wake up-get going (sedentary)**<br>Slumped on the couch<br>Cant really do anything (sit in bed or on the sofa)

Confidential: For Review Only
